# Evolution of heterogeneous genome differentiation across multiple contact zones in a crow species complex

**DOI:** 10.1038/ncomms13195

**Published:** 2016-10-31

**Authors:** Nagarjun Vijay, Christen M. Bossu, Jelmer W. Poelstra, Matthias H. Weissensteiner, Alexander Suh, Alexey P. Kryukov, Jochen B. W. Wolf

**Affiliations:** 1 Department of Evolutionary Biology and Science for Life Laboratories, Uppsala University, Norbyvägen 18D, Uppsala 75236, Sweden; 2 Department of Zoology, Population Genetics, Stockholm University, Stockholm SE-106 91, Sweden; 3 Laboratory of Evolutionary Zoology and Genetics, Institute of Biology and Soil Science, Far East Branch Russian Academy of Sciences, Vladivostok 690022, Russia; 4 Division of Evolutionary Biology, Ludwig Maximilian University of Munich, Grosshaderner Street 2, Planegg-Martinsried 82152, Germany

## Abstract

Uncovering the genetic basis of species diversification is a central goal in evolutionary biology. Yet, the link between the accumulation of genomic changes during population divergence and the evolutionary forces promoting reproductive isolation is poorly understood. Here, we analysed 124 genomes of crow populations with various degrees of genome-wide differentiation, with parallelism of a sexually selected plumage phenotype, and ongoing hybridization. Overall, heterogeneity in genetic differentiation along the genome was best explained by linked selection exposed on a shared genome architecture. Superimposed on this common background, we identified genomic regions with signatures of selection specific to independent phenotypic contact zones. Candidate pigmentation genes with evidence for divergent selection were only partly shared, suggesting context-dependent selection on a multigenic trait architecture and parallelism by pathway rather than by repeated single-gene effects. This study provides insight into how various forms of selection shape genome-wide patterns of genomic differentiation as populations diverge.

## Abstract (web summary)

Genetic changes accumulate as populations diverge and new species emerge. Here, Vijay *et al*. resequence the genomes of crow populations at various stages of genetic differentiation, and shed light on the evolutionary processes acting during the origin and hybridization of crows in Europe and Asia.

## Graphical abstract

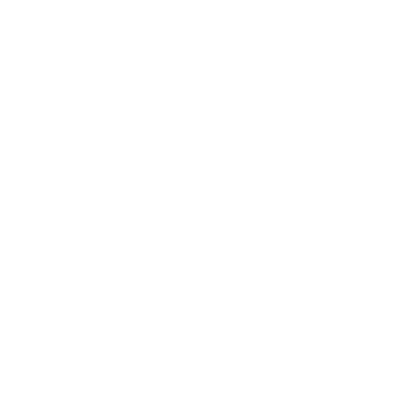

The question of how new species originate and adapt to novel environments has been the foundation to the field of evolutionary biology. Rapid development of high-throughput sequencing technologies during the last decade has opened new avenues to address the genetic basis of adaptation and speciation in natural populations. By studying patterns of genetic variation across the genomes of multiple individuals sampled in their natural environment, evolutionary processes governing the accumulation of genetic differences between incipient evolutionary lineages can be caught in the act^1^,^2^. Recent population genomic studies performed in a variety of taxa share a central theme: as populations diverge, genetic changes accumulate non-randomly across the genome with regions of locally elevated differentiation emerging over genome-wide background levels^3^,^4^,^5^. Building on the idea of a ‘genic model of speciation', regions of elevated differentiation, typically inferred by relative measures of genetic differentiation such as F_ST_, are candidates for being involved in reproductive isolation^6^,^7^. Under this model, allelic variants under divergent selection conferring reproductive isolation (and sites in close linkage) will be less likely to cross population boundaries upon admixture than unlinked, selectively neutral loci^8^. As a consequence they are sheltered from the homogenizing process of gene flow reducing genetic differentiation.

Yet, local peaks of genomic differentiation need not necessarily arise as a result of divergent selection advancing reproductive isolation. There is increasing evidence that genomic regions with elevated differentiation can emerge by processes unrelated to speciation^9^,^10^,^11^. Even in the absence of gene flow, selection either in the form of genetic draft (on loci that may or may not be relevant for speciation)^12^,^13^ or background selection (generally unrelated to speciation)^14^ can locally reduce genetic diversity at linked, neutral sites (‘linked selection'), accelerate lineage sorting, and significantly contribute to heterogeneity in differentiation^11^,^15^.

Discerning evolutionary processes leading to varying levels of differentiation across the genome is a challenging task. A promising way forward is to sample several populations at various stages of population divergence^2^. Using this approach, recent studies have shown that in early stages of population divergence differentiation peaks can be confined to a small proportion of the genome^16^,^17^. As divergence progresses, common background levels of differentiation caused by linked selection on a shared genomic architecture get ever more exposed^4^,^18^,^19^. In addition to temporal multi-population sampling at different stages of the ‘speciation continuum', independent population replication across repeated (often ecological) contrasts has proven powerful to unveil parallel selection pressures contributing to increased local genetic differentiation. For instance, global sampling of stickleback populations across a variety of repeated ecological contrasts revealed recurrent effects of the same major effect genes, but also peculiarities given each genotypic background^20^,^21^.

We here report insights from population comparisons of the *Corvus* (*corone*) spp. crow species complex at various stages of genetic differentiation (F_ST_ range: 0.02–0.47; Supplementary Table 1; *Corvus* (*corone*) spp.=*corone/cornix/orientalis/pectoralis* hereafter). Described in detail almost 100 years ago^22^, the system is characterized by vicariance associated with a phenotypic contrast in plumage colouration (Fig. 1a). Popularized by Mayr in the mid 20th century^23^, it has become a textbook example for incipient speciation involving a sexually selected trait^24^,^25^ that evolved independently in other species of the genus (see supplementary figure 1 in ref. 17). In a geographically alternating pattern, all-black forms (*corone*, *orientalis*) occur both east and west of populations of a black-and-grey pied phenotype (mostly *cornix*), with hybrid zones in Europe and Siberia (Fig. 1a). At the south-eastern margin of the species distribution another pied form occurs, the collared crow *pectoralis,* with a reduced light-coloured dorsal and ventral region (Fig. 1a). Eurasian hybrid individuals show similar variation in the degree of grey plumage from extensive dorsal and ventral patches to narrow rings comparable to the collared crow phenotype in Asia (Fig. 1b). Recent genomic and transcriptomic work across the European hybrid zone (*corone*–*cornix*) has shown that only a small number of narrow genomic regions, associated with the striking difference in pigmentation pattern, exhibited resistance to gene flow and suggests a simple, yet multi-locus genetic architecture for the trait^17^,^26^. Here, we analysed whole-genome re-sequencing data of 124 individuals from 10 populations, including 11 phenotypic hybrids, and 6 individuals from the recently diverged American sister species *C. brachyrhynchos* (mean coverage: 12.5 × ; range: 6.6–26.6; Supplementary Table 2). Exploiting variation in population connectivity ranging from allopatry to ongoing gene flow across multiple contact zones, in combination with the contrast in phenotype, allowed us to characterize the evolutionary processes underlying genomic differentiation during incipient speciation. In brief, genomic regions of elevated differentiation were best explained by selection pressures common to all populations. Superimposed on this background, divergent selection against gene flow was specific to each contact zone and linked to phenotype by metabolic pathway rather than by repeated selection on individual genes.

## Results

### Population structure

**Figure 1:**
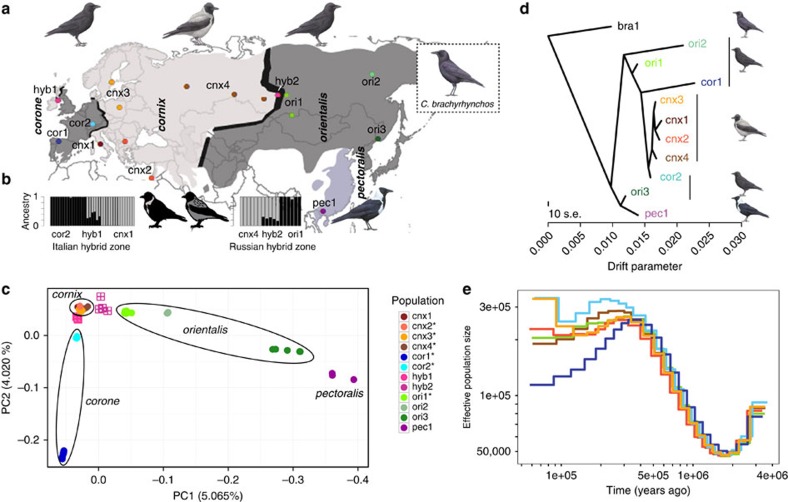
Population structure and demographic history. (**a**) Distribution map of the *Corvus* (*corone*) spp. species complex displaying sampling location, taxon name and phenotype (area shading: light-grey=hooded, grey=collared, dark-grey=all-black). Well-characterized hybrid zones are shown as black solid lines. The all-black American crow *C. brachyrhynchos* is confined to North America. Abbreviations and colours indicating population units are maintained throughout: *cor=C.* (*c.*) *corone; cnx=C.* (*c.*) *cornix*, *ori=C.* (*c.*) *orientalis*, *pec=C.* (*c.*) *pectoralis*, *bra=C. brachyrhynchos.* Crow drawings courtesy of Dan Zetterström. See Supplementary Methods for map details. (**b**) Ancestry coefficients of hybrids and individuals of adjacent parental populations. Genetic clusters are coloured in correspondence to the phenotype of parental populations (black=all-black, grey=hooded). Interspersed are representative images of hybrid phenotypes. (**c**) Main principle component axes partitioning genetic variation of 16.6 million single-nucleotide variants segregating across all populations. PC1 explained 5.0% of the genetic variation mainly separating *corone/cornix*, *orientalis* and *pectoralis*, PC2 (4%) mainly accounted for variation due to the Spanish *corone* population. (**d**) Evolutionary relationships as inferred by TreeMix rooted with the American Crow. (**e**) Changes in effective population size (N_e_) through time as inferred by multiple sequential Markovian coalescent analyses shown for representative individuals from a subset of populations (marked with a star in **c**).

Of a total of 16.6 million single-nucleotide polymorphisms (SNPs) segregating across all populations, 11.8% were shared with the American crow. Superimposed on a weak isolation-by-distance (IBD) pattern across the Palearctic (Mantel test: *r*=0.47, *P*=0.009), population structure was dominated by deep divergence among (black, presumably refugial) Spanish *corone* and eastern Russian *orientalis* populations—neither reflecting subspecific delineation nor plumage colouration (Fig. 1c,d, Supplementary Fig. 1). Recent ancestry with the all-black American sister species (genome-wide F_ST_ 0.27–0.47) supports a monophyletic origin of the Eurasian species complex from a black ancestor in East Asia. Concordant demographic histories suggest shared ancestry of all Eurasian populations followed by divergence during the late Pleistocene (Fig. 1e). Principle component analyses (Fig. 1c), ancestral population graph reconstruction (Fig. 1d), and phylogenetic network analyses (Supplementary Figs 2 and 3) clearly demonstrate an independent origin of both pied subspecies, *pectoralis* and *cornix,* from black ancestors. A recent and common origin of all *cornix* populations is further supported by very shallow population structure (mean genome-wide F_ST_ 0.016–0.031 versus 0.017–0.270 for all other comparisons, Supplementary Table 1) and strongly negative residuals in the IBD correlation (Supplementary Fig. 4).

Overall, this portrays an evolutionary scenario where a single *cornix* population expanded into suitable habitat after the last glacial retreat and came into secondary contact with divergent black populations approximately ten millennia ago^23^. In the European contact zone, isolation-with-migration models suggest asymmetric gene flow from *cornix* into the central European *corone* population^17^. Admixture analyses support genome-wide introgression between hooded *cornix* and both black *corone* to the west and black *orientalis* to the east (Supplementary Fig. 1), while still maintaining phenotypically narrow hybrid zones co-aligning with major suture zones of other biotic assemblages^27^ (Fig. 1a). Hybrids from both the European and Siberian hybrid zones were genetically intermediate between geographically adjacent parental populations (Fig. 1c), had higher levels of genome-wide linkage disequilibrium (LD) than neighbouring parental populations (Kruskal–Wallis test, *P*=0.0308, 0.0063), and showed a range of admixture proportions (0.532–0.886) attesting to ongoing backcrossing (Fig. 1b). Genome-wide introgression across all three contact zones was further supported by significant Patterson's D statistics (Supplementary Table 3). This evolutionary history of the species complex sets the stage to investigate the dynamics of selection maintaining phenotypic integrity in the face of gene flow across multiple contact zones. Note that due to recent shared ancestry of all hooded crow populations contact zones between *corone*–*cornix* and *cornix*–*orientalis* are not fully independent in that both may reflect selection common to all *cornix* populations.

### General patterns and processes of genome-wide differentiation

**Figure 2:**
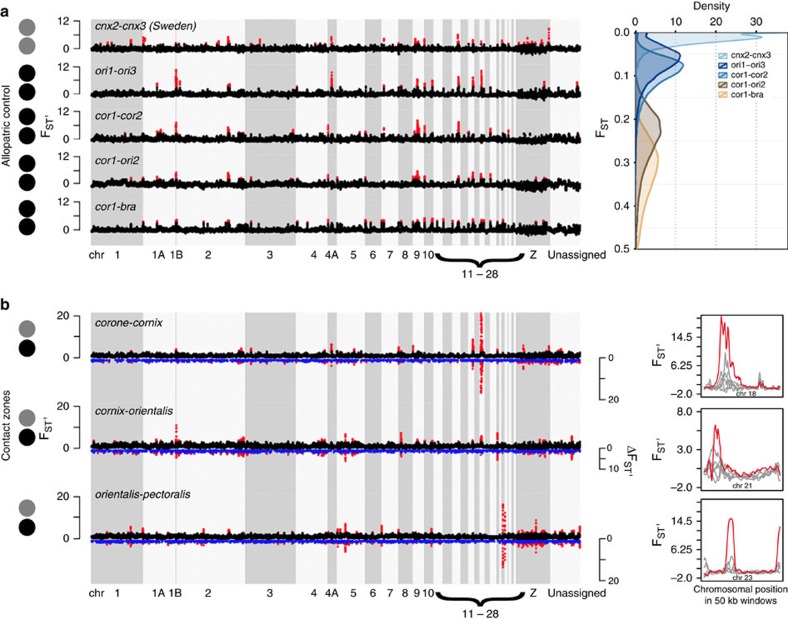
Heterogeneous genomic landscapes of genetic differentiation. (**a**) Left: standardized genetic differentiation F_ST_' in 50 kb windows across the genome between a set of five control population comparisons of the same colour phenotype. The *y*-axis represents s.d.'s of a standard normal distribution. Right: population comparisons span a broad range of absolute genetic differentiation F_ST_. (**b**) Left: standardized genetic differentiation F_ST_' (black, positive axis) and net genetic differentiation ΔF_ST_' (blue, mirrored to the negative axis) in 50 kb windows across the genome between target population comparisons across contact zones (Fig. 1a). Genomic regions of extreme differentiation (>99th percentile) are shown in red for both F_ST_' and ΔF_ST_'. Note that the *y*-axis is drawn to scale for **a** and **b**. Right: examples of outlier regions with the most extreme amplitudes in any of the three comparisons (grey: control, red: target comparisons). Note the different scale on the *y*-axis.

**Figure 3:**
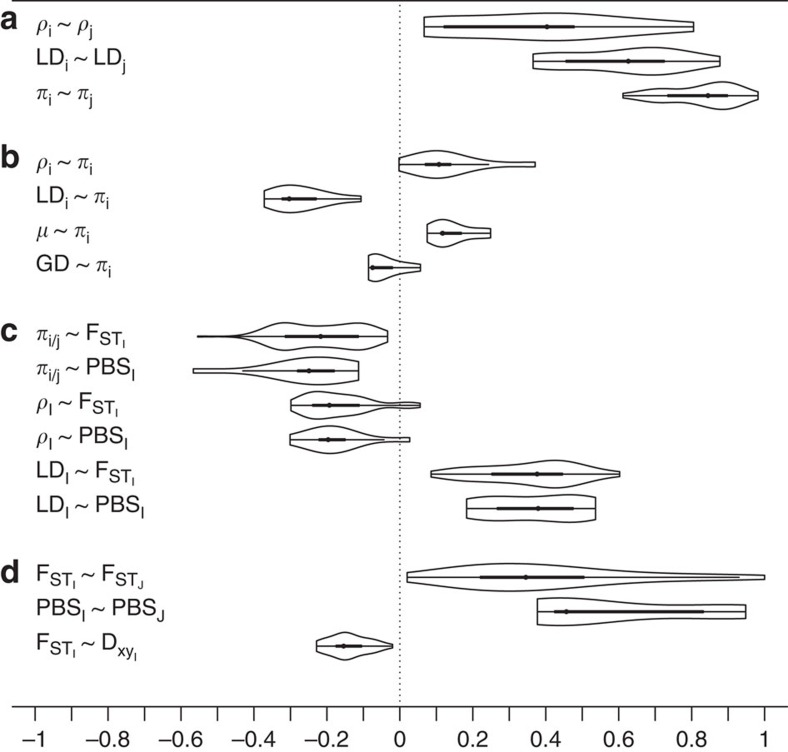
Evidence for linked selection shared among populations. Distribution of correlation coefficients (Pearson's *r*) for population summary statistics shown as box plots with kernel densities drawn on each side. Note that the vast majority of correlations do not overlap zero. Correlations are shown for intra-population summary statistics (**a**) between populations, (**b**) within populations, (**c**) in comparison with inter-population (differentiation) statistics and (**d**) for inter-populations statistics of population pairs (comparing differentiation/divergence landscapes). Box margins indicate the interquartile range between 25 and 75% quantiles, whiskers extend to 1.5-times the interquartile range encompassing 99.3% of the distribution. Subscripts ‘*i*, *j*' symbolize all possible combinations between two populations *i=1...n* and *j=i+1....n* for within-populations measures; Capital letters ‘*I*, *J*' symbolize inter-population statistics. Correlations exclude pseudo-replicated population comparisons (for example, *I*=ori1,cnx1, *J*=ori1,pec).

Strong heterogeneity in genetic differentiation as measured by F_ST_ and F_ST_' (z-transformed F_ST_ reflecting genome-wide variance of F_ST_ in terms of s.d.'s) for 50 kb windows across the genome was observed for all 54 population comparisons (Fig. 2a,b). Windows with elevated levels of differentiation (>99th percentile, F_ST_') were significantly clustered into distinct ‘outlier peaks' with mean sizes ranging from 117 to 295 kb (range max. size: 200–1,800 kb, *p*_all_<0.001). With progressing levels of overall differentiation, peaks crystallized more clearly, as evidenced by an increase in autocorrelation (Moran's *I*) between windows and co-variation in differentiation profiles (Fig. 2b, Supplementary Fig. 5). This points at parallel, long-term processes acting across the genome with a prime role of linked selection common to all populations and/or their most recent common ancestor. This was further supported by the following evidence summarized in Fig. 3 (see also Supplementary Table 4):

First, genome-wide profiles of the population recombination rate *ρ* (=4*N_e_r*) and LD were positively correlated ‘among' all possible population pairs supporting the assumption of broad-scale recombination rate conservation expected across this short evolutionary timescale; and hence fulfilling a central precondition for shared linked selection across populations.

Second, consistent with a role of shared linked selection in reducing polymorphism, nucleotide diversity *π* was correlated ‘among' populations, even when controlling for the effect of *μ* (approximated by synonymous substitution rate). ‘Within' populations, *ρ* (and resulting LD) correlated strongly with *π* (=4*N_e_μ*). With little evidence for recombination-associated mutation (and hence *r*∼*μ*) (ref. 15) and *ρ* generally recapitulating recombination rate^28^, this provides evidence for variation in effective population size (N_e_) along the genome being reduced in regions of low recombination. *π* was most reduced in low recombination regions associated with a high density for targets of selection measured as the density of coding sequence, a signature expected for background selection (positive statistical interaction *ρ* × gene density significant in 8 out of 10 populations, Supplementary Table 5)^14^. This genome-wide interaction corresponds to a central prediction of background selection^15^, which has also been suggested as a key process reducing overall diversity in species with similar effective population sizes^19^,^29^.

Third, measures of genetic differentiation were negatively correlated with *π* and *ρ* (and positively with LD). This was true for F_ST_ between population pairs, but also for lineage-specific differentiation as estimated by the population branch statistic (PBS) removing effects due to shared ancestry and pseudo-replication. While a correlation with *π* is expected by the fact that F_ST_ is a relative differentiation measure sensitive to population diversity, a correlation with *ρ* is not expected under neutrality and requires the assumption of linked selection locally reducing effective population sizes in regions of low recombination.

Fourth, differentiation landscapes co-varied positively both for F_ST_ among all population pairs and for lineage-specific PBS estimates. Moreover, F_ST_ co-varied negatively with net divergence between populations (*D*_xy_) suggesting that linked selection acted already in the ancestral population^11^.

In line with theoretical predictions^14^,^15^ and empirical evidence^19^,^30^ these findings are best explained by a common genomic architecture exposing the effects of linked selection.

### Evolutionary processes acting across contact zones

To isolate genomic regions subjected to selection acting specifically at contact zones with phenotypic transitions (Fig. 1a), we took a comparative approach. We defined a set of five allopatric population comparisons matched by phenotype and spanning a gradient of overall genomic differentiation (Fig. 2a). These comparisons constitute a null model for shared heterogeneity in differentiation arising through processes unrelated to reproductive isolation in the absence of (recent) gene flow. Maximum F_ST_' from any of these five comparisons is taken as a conservative estimate of the strength of linked selection. Subtracting maximum F_ST_' from orthologous windows of the focal comparisons (Fig. 2b), we accordingly obtained a statistic of net differentiation (ΔF_ST_') measuring relative excess differentiation in s.d. units. Windows classified as outliers (>99th percentile) for F_ST_', but not ΔF_ST_', were interpreted as genomic regions subject to shared selection pressures (‘shared peaks'; Supplementary Table 6), whereas windows classified as outliers for both statistics potentially result from selection pressures specific to one or more phenotypic transitions (‘contact zone peaks') exceeding background levels of shared, linked selection. In several populations, contact zone peaks carried significantly stronger signatures of selection compared with windows lost from ‘shared peaks' after subtraction: *π* was reduced, Fay and Wu's H remained reduced, and LD, haplotype length (iHH), and lineage-specific differentiation (PBS, F_ST_') were elevated in at least one of the contact zones (Supplementary Table 7) exceeding levels seen in any allopatric control comparison. The strength of selection signatures of ‘contact zone peaks' differed among contact zones. Signals specific to the Siberian contact zone did not differ significantly form ‘shared peaks' suggesting selection to be weaker or more ancient than in the European zone. Indeed, under the assumption that the crow hybrid zone constitutes a tension zone with a constant median dispersal distance *σ*, 9- to over 200-fold lower selection coefficients are expected in the three to sevenfold wider Siberian hybrid zone^31^ (according to *s∼*(*σ/w*)^2^) (ref. 32).

**Figure 4:**
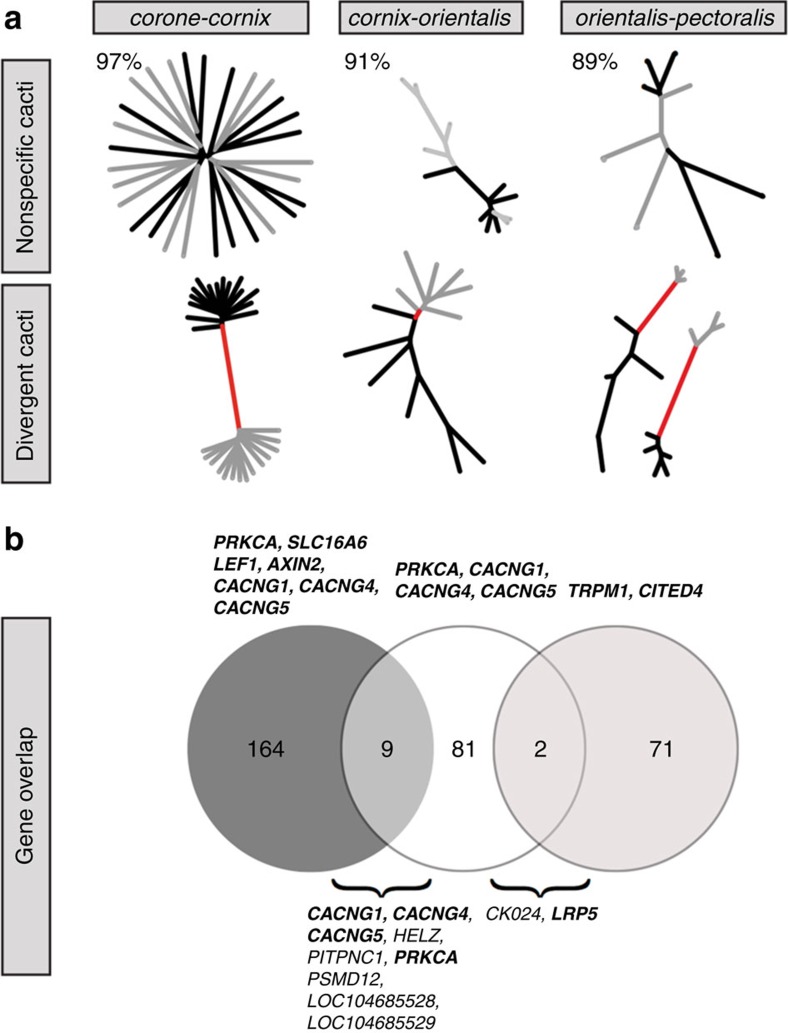
Localized phylogenetic patterns and candidate gene overlap. (**a**) Illustration of HMM-SOM local phylogenetic reconstructions (‘cacti') across each of the target zones of contact and phenotypic transition. Most of segregating variation across the genome (indicated in percentage) is represented by ‘non-specific cacti' holding no information on population provenance (grey tips: hooded phenotype, black tips: all-black crows). Much rarer ‘divergent cacti' separate individuals by population and phenotype (red branches). (**b**) Venn diagrams illustrating the number of candidate outlier genes for each specific contact zone (in the same order as in **a**) and the degree of overlap between population pairs. Melanogenesis outlier genes are listed for the contact zones; for the overlap all genes are listed by name with those involved in melanogenesis shown in bold.

Focusing on genes located in ‘contact zone peaks', in genomic regions flagged by divergent local phylogenies (‘cacti', Fig. 4a), and those containing fixed SNPs (gene+10 kb vicinity; Supplementary Table 8), we assessed the degree of parallelism in genes potentially under selection within and across the three contact zones.

We first focused on the *corone*–*cornix* contact zone. Parallel evolutionary dynamics along the European hybrid zone were indicated by congruent differentiation landscapes across the central and southern part of the hybrid zone, Germany with Poland/Sweden (re-analysed from ref. 17) and with Italy (this study), respectively (Supplementary Fig. 6). Correlations were high for F_ST_ (*r*=0.74) and even higher for ΔF_ST_' (*r*=0.94), suggesting a stronger signal for peaks associated with gene flow across the hybrid zone. In all, 58% of all flagged genes were shared and both comparisons contained the same set of melanogenesis-related genes in a major >2 Mb peak on chromosome 18 (for example, *CACNG1/4/5*, *AXIN2*, *PRKCA*, Fig. 4b). An additional pigmentation candidate, *LEF1*, a transcriptional regulator acting within the Wnt signalling pathway, was identified on chromosome 4 for the southern part of the zone. Outlier genes were enriched for GO categories ‘calcium channel activity', ‘calcium ion transport' and ‘calcium ion transmembrane transport'. No enrichment was found for any KEGG pathway (Supplementary Table 9).

Examining the extreme values of the haplotype statistics nSL, iHS and XP-EHH (<0.05 and >99.5 percentile, respectively), additional loci potentially affected by positive selection were identified. Importantly, outlier genes with described functionality in the melanogenic pathway such as *CACNG5*, *AXIN2* or *PRKCA* or visual perception such as RGS9 flagged by window-based and localized phylogenetic were supported by SNPs with extreme standardized haplotype statistics.

Of all contact zones, outlier windows in the Siberian *cornix*–*orientalis* hybrid zone were least clustered, formed smaller contiguous peaks (Fig. 2b, Supplementary Table 10), and showed weaker signals of divergent local phylogenies (Fig. 4a).

Only two moderately sized ΔF_ST_' peaks (both 350 kb) with low relative amplitudes emerged, on chromosome 21 and the Z-chromosome (Fig. 2b). Of a total of 35 fixed differences, a single one was located within an exon, in the gene *LRP5*. Altogether with *LRP6*, located in a 50 kb outlier window, this gene is a key component of a co-receptor group acting within the Wnt signalling pathway. It interacts closely with *AXIN* genes^33^ found in the main differentiation peak in the European hybrid zone^5^. *LRP6* and *LRP5* genes also contained several segregating sites with extreme values of haplotype statistics signalling the footprint of positive selection. Two intronic variants in *LRP6* were detected in all haplotype outlier scans, however *LRP5* was only identified as an outlier in nSL and XP-EHH, suggesting a possible soft sweep of this gene in the *orientalis* population. Overall, outlier genes were not enriched for any GO category or KEGG pathway.

In the *orientalis*–*pectoralis* contact zone, similar to *corone*–*cornix* in Europe, the differentiation landscape was dominated by one prominent ΔF_ST_' peak spanning over 800 kb on chromosome 23 (Fig. 2b). It contained 16 genes, 1 of which, *STX12,* affects pigmentation by involvement in melanosome trafficking^34^. *LRP5*, described above for the *cornix*–*orientalis* contact zone, was located in an outlier window and also associated with divergent cacti (Fig. 4a). The selection signal of XP-EHH, iHS and nSL, supported positive selection of *LRP5* in both populations, however, only intra-population haplotype statistics point to selection in *STX12.* Multiple variants with a positive selection signal were located in intronic regions of *LRP5* and *STX12*, however, three selected outlier variants were located in exon 8 of *STX12*. Overall, outlier genes were not enriched for any GO category or KEGG pathway.

Across contact zones, correlations of differentiation metrics were weak (F_ST_: *r*=0.03–0.15) compared with allopatric controls (*r*=0.16–0.67; Supplementary Table 11), outlier windows hardly overlapped (range: 0–3%), and not a single candidate gene involved in pigmentation was shared across all three comparisons. Nonetheless, a small number of candidate genes were shared between contact zone pairs, suggesting context-specific selection on an oligo-genetic molecular trait architecture (Fig. 4b). Outlier genes shared by *corone*–*cornix* and *cornix*–*orientalis* comparisons were enriched for nine GO categories, all related to calcium and ion transport (Supplementary Table 9) consistent with the previous implication of voltage-dependent calcium channel subunits in pigmentation shift in the central European hybrid zone^17^. Enrichment of shared outlier genes for the KEGG pathways ‘MAPK signalling pathway' and ‘cardiac muscle contraction' (Supplementary Table 9) was similarly explained by the presence of previously implicated genes, *CACNG* genes and *PRKCA,* associated with both pathways.

While no candidate gene was shared among all contact zones based on the F_ST_ based outlier scan, haplotype-based analyses highlighted 93, 100 and 257 SNPs located in genic regions (for iHS, nSL and XP-EHH, respectively) that were shared across all contact zone comparisons. While caution is warranted for single SNP outlier tests, this number significantly exceeded the random expectation of flagging the same site in three contact zones (0.01^3^ × total SNPs considered). Within this shared selected gene set, six genes had a functional link to the melanogenic pathway, including *TRPM1*, *KCTD5*, *MMP17* and *INPP4B* detected by nSL, *GREB1* identified with XP-EHH and *FREM2* detected by iHS.

More generally, pigmentation candidate genes from across all contact zones contained at least one gene associated with the Wnt signalling component of the melanogenesis pathway (*AXIN2*, *LEF1*, *LRP5/6*), a central modulator of the microphthalmia-associated transcription factor (*MITF*) that has been functionally implicated in the phenotypic differences between European crows^26^.

## Discussion

Our results suggest parallelism by pathway rather than by repeated single-gene effects^35^. This contrasts with findings in *Heliconius* butterflies where the same loci, associated with similarly few and extreme outlier regions, code for mimicry colour phenotypes across replicate hybrid zones^36^. The isolation of few loci specific to each contact zone also contrasts with studies implicating a high number of prominent outlier regions hosting candidates under divergent selection associated with the (ecological) contrast in question^37^,^38^. The apparent difference in number and size of differentiation peaks begs the question on the genetic architecture underlying the trait under selection. Strong, recent selection on traits with a presumably simple (mono- or multigene) genetic architecture such as colour pattern in *Heliconius* or crows are expected to rapidly shift population allele frequencies and thereby increase local genetic differentiation at one or few causal loci and linked neutral sites. Selection on traits encoded by many genes, however, should in principle not be detectable in genome scans, which are effectively blind to selection on small-effect polygenes, or when epistasis is involved^39^. The few and strong candidate selection signals observed in this study suggest a simple genetic architecture of the trait under selection. The fact, however, that peaks of elevated differentiation emerged in non-orthologous genomic regions in different population comparisons similarly implies a multigenic architecture—consistent with the variable segregation pattern of phenotypes in the hybrid zone. Differences in the genic target among populations may in part arise due to unique, local genetic variation on which selection can act, and in part due to different upstream modulators affecting phenotypic differences between *cornix* and *pectoralis*. Explaining the pervasive heterogeneity in genomic differentiation observed across many taxa thus remains a major challenge for speciation genomic research.

In the last decade, there has been much enthusiasm on finding ‘speciation genes' in genomic regions of elevated differentiation. As the field has matured, it is increasingly being recognized that local peaks of genomic differentiation do not necessarily arise as a result of divergent selection on allelic variation reducing gene flow and advancing reproductive isolation^11^,^19^,^30^. The results from this study illustrate that heterogeneous genomic differentiation emerges largely through linked selection shared among all populations across the ‘speciation continuum'^2^. Yet, superimposed on these common processes, we identified genomic regions with signatures of selection against gene flow, specific to independent phenotypic contact zones. Candidate pigmentation genes with evidence for divergent selection were only partly shared suggesting a contribution of context-dependent divergent selection by metabolic pathway in maintaining phenotypic integrity against gene flow at secondary contact zones. The study thus highlights the importance of decomposing shared and population specific components of genetic differentiation among populations to infer evolutionary process from ‘disputed islands of genetic differentiation'^40^.

## Methods

### Population sampling

We sampled a total of 107 morphologically pure individuals of the Eurasian *Corvus* (*corone*) spp. species complex from populations across its geographical distribution (spp. referred to by their (sub)species designation *corone/cornix/orientalis/pallescens/pectoralis*) and sampled 11 hybrids from within the European and Siberian hybrid zones (Fig. 1). We further obtained samples for six individuals of the American sister species *Corvus brachyrhynchos.* Inclusion of the American crow constitutes an important basis for an empirical null distribution of heterogeneous genome-wide differentiation in the absence of recent gene flow (see Supplementary Notes). Two individuals each from rook (*Corvus frugilegus*) and Eurasian jackdaw (*Corvus monedula*) were added as outgroup taxa. Information on taxonomic status of the species complex, and details on samples and sampling locations are given in the Supplementary Notes and Supplementary Table 2.

Where possible, (homogametic) male individuals were chosen to guarantee equal sequencing coverage for autosomes and the Z-chromosome, and to avoid systematic biases arising due to coverage differences for population genetic analyses. Yet, due to sample limitations, a total of 23 (out of 118) heterogametic females were included in the *Corvus* (*corone*) spp. population samples and three females out of six American Crows. For details on sex determination see Supplementary Notes.

### Data generation and basic processing

#### Whole-genome re-sequencing

We extracted genomic DNA using either a standard phenol-chloroform protocol or the Qiagen DNeasy blood and tissue kit. Subsequently we prepared one paired-end library per individual (average insert size: 400 bp, s.d.: 28 bp) with Illumina TruSeq DNA Sample Prep Kit v2 according to the manufacturer's instructions. All libraries were sequenced on an Illumina HiSeq2000 machine. Average raw read sequence coverage including the published data was 12.5 × (range 6.6 × to 26.6 × , first/third quantiles: 9.7/17.5 × ) assuming a genome size of 1.21 Gb (see ref. 17 and references therein). Individually barcoded libraries were split into five aliquots each and distributed across lanes and flow cells in a random blocked design. Coarse initial quality assessment of all re-sequenced data was performed with the fastQC toolkit (http://www.bioinformatics.babraham.ac.uk/projects/fastqc).

Two of the *pectoralis* samples were obtained from toe pads of museum specimens collected during the 1920s. Sequencing libraries for these samples were directly produced from DNA extractions without further fragmentation. To assess the potential contribution of *post mortem* DNA damage which could confound the population genetic analyses, we quantified cytosine deamination at read ends using PMDTools^41^. Visual inspection of the frequency distribution of PMD scores did not reflect any differences between museum specimens and freshly collected samples, suggesting no substantial *post mortem* DNA degradation.

#### Variant discovery and genotyping

Raw reads were mapped to the hooded crow reference assembly^17^ available at the National Center for Biotechnology Information (NCBI) under Genbank accession number JPSR00000000. Read mapping was performed with the Burrows-Wheeler Aligner *bwa 0.7.8* (ref. 42) with default settings. The *picard* software (http://broadinstitute.github.io/picard) was subsequently used to assign readgroup information containing library, lane and sample identity. To enhance the alignments in regions of insertion–deletion polymorphisms, we additionally performed local realignment using *GATK*^43^ before duplicate read-pairs were marked at the library level with *picard. Bam* files were merged at the individual level, validated, and duplicate reads removed in *picard* before variant discovery.

Variant discovery was performed for the 118 crow samples including hybrids together and separately for the 6 American crow samples using *HaplotypeCaller* in *GATK* version 3.3.0. Variant detection on autosomal regions utilized one group comprised of all male and female samples under the diploid default setting (−*ploidy* 2). Z-linked chromosomal regions were analysed separately for male and female groups, by specifying the ploidy level for male samples as ‘−ploidy 2' and for heterogametic female samples as ‘−ploidy 1'.

Base quality score recalibration is known to improve variant discovery and genotype calls, but requires knowledge of true variant sites. Here, we intersected the reliable set of high-quality SNPs analysed in Poelstra *et al*.^17^ with an initial round of variant calling on the original, uncalibrated sequence alignment files using *HaplotypeCaller* in *GATK* version 3.3.0. using default settings. Variant quality score recalibration, a post-discovery error modelling algorithm implemented in *GATK*^43^ can further improve variant calling. In the absence of previous knowledge of ‘true' variants, we utilized 10–15% variants with the highest genotype quality scores to generate an error model. Finally, a catalogue of all variable sites within and between all hooded and carrion populations was subsequently genotyped using *GATK*. In a final filtering strategy, variants within regions with annotated repeat content were removed (see Supplementary Notes for repeat annotation).

#### Ancestral state reconstruction

Several population genetic statistics require information on the ancestral state of segregating variants. We reconstructed the ancestral state for each segregating site in the genome as described in Poelstra *et al*.^17^.

#### Haplotype phasing

Phasing was performed using fastPHASE^44^. For each scaffold, fastPHASE was run using all 118 Eurasian crow individuals including hybrids, with population labels provided using the *−u* option. Since many populations contained heterogametic females reducing effective sample size in sex chromosomes, phasing was limited to autosomes.

### Population structure and gene flow

#### Mitochondrial network analysis and phylogeny

Consensus sequence of mitochondrial genomes was extracted from a subset of individuals specified in Supplementary Table 2 retaining only those individuals that had <200 sites with missing or ambiguous data. Haplotype networks were constructed with Network 4.6.1.3 (available at www.fluxus-engineering.com; Fluxus Technology Ltd.) using the Median Joining method based on the maximum-parsimony algorithm^45^.

In addition to mitochondrial network analyses, we reconstructed phylogenetic trees from 81 complete *corone/cornix/orientalis/pectoralis* crow mitochondrial genomes using maximum-likelihood procedures (for details see Supplementary Notes).

#### Split decomposition network

To investigate the evolutionary relationships among the *Corvus* (*c.*) spp. complex, we utilized a network reconstruction method that incorporates incompatible and ambiguous signals inherent within a genome. A split decomposition network was reconstructed using the program *SplitsTree* on the autosomal variant data set^46^, first including 107 individuals (removing hybrids), and subsequently focusing on the Russian populations and *pectoralis.*

#### TreeMix

We further used a statistical model by Pickrell and Pritchard^47^ implemented in the program Treemix to infer relationships between populations by estimating population splits based on their genome-wide allele frequency distributions. The program was run using a window size of 1,000 SNPs (results with window sizes of 100, 200 and 500 had the same tree topology) and was rooted with the American crow (*Corvus brachyrhynchos*).

#### Principle component analysis

Principle component analysis was performed on all SNPs following the approach by Patterson *et al*.^48^ as implemented in the software *Eigensoft*, which pruned the initial variant set to 16,064,921 SNPs.

#### Isolation by distance

We quantified the relationship between geographical and genetic distance (measured as F_ST_/1-F_ST_) among populations. For population pools from different sampling locations (cnx3, cnx2, cnx4, ori1), the average longitude and latitude values weighted by sample size were used as geographic location. Population pairs with high negative residuals were taken to be genetically more similar than expected by geographic distance as across the entire species complex. A Mantel test was used to assess statistical significance of the correlation between geographic distance and genetic distance, while controlling for pseudo-replication among all possible population comparisons. In the main text, we report the correlation for the entire nuclear genome; correlations for sex chromosome and autosomes were near identical (*r*=0.47/0.46, *P*=0.007/0.008). For details on analytical procedures see Supplementary Notes.

#### Ancestry estimation

We first performed admixture analyses as implemented in the software program *ADMIXTURE version 1.23* (ref. 49) on the set of 107 phenotypically pure individuals from the C. (*c.*) spp. complex. Analyses were run on a total of 15,937,359 segregating autosomal sites, including only variants with <10% missing genotypes. We investigated between 1 and 10 possible population clusters, performing 200 bootstrap resampling iterations to estimate the s.e.

In addition, we calculated admixture proportions of the six European hybrid individuals and five Russian hybrid individuals relative to the surrounding parental populations (Europe: cor2–cnx1, Russia: cnx4–ori 1, see Fig. 1). We calculated admixture proportions both for the entire genome-wide set of variants and a subsample of segregating variants from within highly differentiated genomic regions between parental populations as inferred by elevated levels of F_ST_ (window-based 99th percentile peak regions, see below), each including only variants with a minor allele frequency greater than 5%. Only the latter data set converged on the expected clustering signal of *K*=2, and accurately assigned the hybrid individuals as admixed. The other data sets likely swamped the diagnostic signal with the overwhelming number of lowly differentiated background variants. Results are therefore reported for the subset of 20,549 and 29,072 SNPs from highly differentiated regions between the parental populations in the Italian and Russian hybrid zone, respectively (Fig. 1).

#### ABBA-BABA test

To test for gene flow between our three focal population comparisons across morphological contact zones we used the four taxon ‘ABBA-BABA' test. The D-statistic as defined in the study by Durand *et al*.^50^ and implemented in the software ANGSD^51^ provides a quantitative test statistic of whether the ABBA or BABA pattern is in excess; a *Z*-score value of 3 and above was considered significant^52^. One high coverage individual was selected as a representative of each population (results with different individuals were qualitatively comparable). As an outgroup the ancestral sequence from three species in the genus *Corvus* was used (see above).

### Demographic history

The multiple sequentially Markovian coalescent approach^53^ was used to estimate historical effective population sizes through time on individuals with at least 20 × mean sequencing coverage. To convert scaled times to years, we used a mutation rate of 3.18 × 10^−9^ per generation and a generation time of 5.79 years (for details see Supplementary Notes).

### Intra-population summary statistics

#### Site frequency spectrum and associated summary statistics

For each population we estimated the unfolded site frequency spectrum (SFS) using the Bayesian method implemented in the software package *ANGSD version 0.588* (refs 51, 54). We included only sites with a minimum mapping quality of 1 and minimum base quality of 20 that were covered in at least three individuals with a minimum depth of four reads each. Using default parameters and genotype likelihoods based on the *GATK* genotyping model implemented in ANGSD, we estimated the unfolded SFS for the whole genome and derived the following summary statistics as averages across 50 kb windows^55^: population mutation rate *θ* (Watterson's estimator), nucleotide diversity (π), Tajima's D, Fay and Wu's H, and Fu and Li's D. As the ancestral reference genome sequence we used the consensus sequence from jackdaw, rook and American crow as described above.

#### Linkage disequilibrium

To estimate genome-wide LD, we divided the phased scaffolds into 50 kb windows corresponding to the coordinates of the window-based analyses. We excluded scaffolds shorter than 50 kb (to correct for physical distance) and scaffolds inferred to be sex-linked due to the expected difference in recombination in sex chromosomes. We extracted SNPs with a minor allele frequency above 0.1 and calculated the mean *r*^2^ value per window, averaging over all pairwise SNP combinations. All filtering and calculation of LD was performed with vcftools^56^. Since *r*^2^ is influenced by sample size (threefold difference between 3 and 15 individuals sampled, see Supplementary Table 12), absolute values are only reported for populations of hybrid zone 1 and 2, sampling 5 and 6 individuals per population for each hybrid zone, respectively.

#### Population recombination rate. *ρ*

To generate an estimate of the population-scaled recombination rate *ρ* per 50 kb window we used LDhelmet^57^. vcftools^56^ and plink^58^ were used to tailor the phased genotype data (see above) to the necessary input file format. These files were concatenated to generate one haplotype configuration file per population in the ‘find_confs' step in LDhelmet. Thereafter, the likelihood lookup tables were computed incorporating the respective estimation of θ, the population-scaled mutation rate (for estimation of θ see above). We also included the optional ‘pade' component of LDhelmet in the analysis, which computes the Padé coefficients from the haplotype configuration file. Then ρ was estimated for every 50 kb window with default parameters (burn-in: 100,000 iterations, MCMC chain: 1,000,000 iterations, block penalty: 50). The output provides a table of estimates of *ρ* per bp for the SNPs in every window, from which we then calculated the weighted mean. We approximated the required mutation matrix from zebra finch substitution rates following Singhal *et al*.^59^. We also used the substitution matrix for chicken^60^ for a subset of the genome where we expected large variance in *ρ* as inferred from LD estimates (scaffold_78 with the main F_ST_ peak in the Eurasian population). Both matrices yielded qualitatively the same results (Pearson correlation of *ρ* estimates *r*=0.99).

### Inter-population summary statistics

#### F-statistics

We calculated hierarchical F-statistics as implemented in the *HierFstat R* package^61^ for all possible pairwise comparisons within the species complex and 7 comparisons with the American Crow (Supplementary Table 1). For each pairwise comparison, F_ST_ was estimated for each segregating site of sufficient sequence coverage (genotypes found for ≥50% individuals per population in each comparison). Autosomal and Z-linked variants were treated separately. Before merging male and female variants on Z-linked scaffolds, male diploid calls were haploidized. Window-based F_ST_ estimates in increments of 50 kb windows across the genome were then calculated by averaging the variance components across all segregating sites within that window.

To assess robustness of genotype-based F_ST_ estimates from *HierFstat*, we additionally used methods specifically designed for low-to-medium-coverage sequencing data yielding highly correlated estimates (Pearson's correlation coefficient: range *r*=0.81–0.93; for details see Supplementary Notes).

#### Population branch statistics

In addition to pairwise F_ST_ estimates we calculated the PBS^62^ for each of the population-specific branches using the formula PBS_Pop1=(−log(1-F_ST_(Pop1_Pop2)))+(−log(1-F_ST_(Pop1_Pop3)))−(−log(1-F_ST_(Pop2_Pop3)))/2.

#### D_xy_

Custom scripts were used to calculate the average number of nucleotide substitutions (*D*_*xy*_) (ref. 63) on the basis of single segregating variants averaged to obtain window-based estimates. Accurate estimates of *D*_*xy*_ require standardization by the total number of available sites per window passing the initial quality filters (that is, not within repeat-masked regions and identified in a minimum of 50% individuals per population). We therefore applied SAMtools ‘mpileup'^64^ to recalibrated ‘.bam' files using the same quality filters for non-segregating sites as for segregating sites.

#### Haplotype statistics

We calculated haplotype statistics reflecting the decay of LD for the adjacent populations spanning each of the three contact zones to infer recent signatures of selection^65^,^66^. Intra-population haplotype statistics include the extended haplotype homozygosity (EHH), the integrated EHH (iHH) and the iHS statistic, the latter providing a measure of how unusual the haplotypes around a given SNP are, relative to the genome as a whole. We further calculated nSL, a similar statistic to iHS, but more robust given uncertainty in the underlying recombination landscape^67^. For comparisons of haplotype length between populations we calculated XP-EHH. The statistics iHS, nSL and XP-EHH were calculated using the program selscan with subsequent downstream standardization of values given 100 allele frequency bins^68^. Since sex chromosomes were excluded from phasing (see above) haplotype statistics are limited to autosomes. Absolute avearge of normalized iHS and nSL across 50 kb windows were taken to compare signals of selection within peak regions with background genomic levels. In addition a single SNP outlier approach was taken, choosing the most extreme haplotype site-specific values (<0.05 and >99.5 percentile, respectively) to focus on particular SNPs in genic regions. The expected number of SNPs to be identified as common outliers for all three hybrid zones by chance is given by (0.1 × prop_hyb1)(0.1 × prop_hyb2)(0.1 × prop_hyb3)( × no. of shared SNPs), where prop_hyb refers to the proportion of outlier SNPs in any of the two populations of a given hybrid zone falling within the subset of SNPs shared among all hybrid zones. For example, we combined the outlier variants for nSL for populations from each contact zone (*corone*–*cornix, cornix*–*orientalis, orientalis*–*pectoralis*) to determine what percentage of outlier loci were in the shared set of SNPs between all three contact zones. In the populations bordering the *corone*–*cornix* contact zones all 78,542 outlier variants (100%) also segregated across all contact zones, while only 59,240 (69%) and 40,233 (48%) in *cornix*–*orientalis* and *orientalis*–*pectoralis* zones, respectively. This yields an expected number of outliers shared among all populations as 0.1 × 1 × 0.1 × 0.69 × 0.1 × 0.48 × 5,447,980∼1.8.

### Substitution rate estimation

We estimated substitution rate at fourfold degenerate sites as a proxy for mutation rate to assess to potential impact of mutation rate on patterns of genetic differentiation. Substitution rate estimates were based on a total of 5,012 reliable 1:1:1: gene-alignments between coding regions of canonical transcripts of chicken (*Gallus gallus*), and collared flycatcher (*Ficedula albicollis*) and crow (*Corvus* (*corone*) *cornix*). For each 50 kb window, substitution rate estimates were averaged across all genes present in that window. The mean genome-wide substitution rate (dS) was 0.079. Substitution rates did not differ between regions of elevated differentiation between taxa (‘peaks' see below, d*S*=0.10) and the genome-wide average in regions outside ‘peaks' (d*S*=0.079, Kruskal–Wallis *P*-value=0.056). See Supplementary Notes for methodological details.

### Outlier screens

To isolate candidate genomic regions under divergent selection refractory to gene flow, we followed the basic logic of traditional genome scans screening for signals of elevated genetic differentiation^69^ on the basis of non-overlapping windows of predefined size. We calculated summary statistics for non-overlapping windows of size 5, 10 and 50 kb resolution. The two smaller sizes generated noisy signals likely flagging substantial amount of false-positive regions (see Supplementary Notes, Supplementary Table 14). Following Poelstra *et al*.^17^ we therefore only report results for 50 kb windows in the main text reflecting stable broad-scale genomic patterns. In addition to window-based approaches we used HMM-SOM method implemented in Saguaro^70^ to identify local phylogenetic relationships across each of the target zones of contact and phenotypic transition (cor2–cnx1, cnx4–ori1, ori3–pec1). We finally considered individual SNPs that appeared as fixed given our sample size. Details on all three approaches, window-based, local phylogenies and SNP-based can be found in the Supplementary Notes. For the window-based analysis we took a comparative, empirical approach to infer evolutionary process from the heterogeneous pattern of F_ST_ contrasting the genomic F_ST_ profiles of focal populations to a set of five control comparisons. Our focal comparisons were between populations with evidence for gene flow and/or transition in pigmentation phenotype including the two Eurasian hybrid zones and the East Asian contact zone between *orientalis* and *pectoralis* (see Fig. 1). Five geographically distant population comparisons of the same phenotype spanning a large range of mean genome-wide F_ST_ were chosen as the control (Supplementary Table 13).

This approach established an empirical null model of differentiation across the genome. It allows assessing whether outlier peaks were most likely driven by processes unique to the focal comparisons (for example, divergent selection against gene flow) or by common selection pressures shared among all population comparisons (for example, background selection,)

In a first step, we determined outlier windows for all controls at the 99th percentile of the Z-transformed F_ST_ distribution (F_ST_') expressing genome-wide differentiation in units of s.d. relative to the mean (Supplementary Fig. 7). On this basis we quantified number of peaks and peak width (in number of adjacent windows). These peaks can be regarded as background heterogeneity arising through processes other than divergent selection across hybrid zones. We then characterized outlier windows of the focal comparisons in the same way. In addition, we subtracted the maximum value of orthologous windows in the controls from each of the focal comparisons (Fig. 2) and determined outlier windows at the 99th percentile for this statistic (called ΔF_ST_' hereafter). Windows classified as outliers for F_ST_', but not ΔF_ST_', are interpreted as genomic regions subject to selection pressures shared across the entire species complex (and likely present in the ancestral population ) independent of specific evolutionary processes acting on any of the target populations. Windows classified as outliers by both approaches were considered to be unique to each focal comparison in genomic position (no peak in controls, but peak in focal population) and/or relative amplitude (also peak in outlier, but with comparatively lower standardized peak height). These ‘unique' outliers were investigated in more detail for gene content, and were contrasted to background genome-wide non-peak regions as well as common peaks for a set of informative summary statistics such as nucleotide diversity (*π*), net nucleotide divergence (*D*_*xy*_), Fay and Wu's H, and haplotype statistics (*r*^2^, iHH, iHS, nSL, XP-EHH; Supplementary Tables 6 and 7).

### Gene ontology/enrichment analyses

We tested whether genes located in genomic windows identified as (F_ST_' and ΔF_ST_') outliers, and genes in regions of divergent cacti, were enriched for particular classes of genes. For gene ontology and KEGG pathway analysis, we first identified chicken orthologs for which gene ontology and KEGG annotations exist, and subsequently tested for enrichment using the goseq R/Bioconductor package^71^. The KEGGREST package^72^ was used to extract KEGG pathway annotations for zebra finch genes (other annotations were natively supported by the goseq package). We also tested for enrichment of melanogenesis-related genes specifically using a manually curated list of melanogensis genes^26^,^73^.

### Data availability

All sequencing data were deposited in the sequence read archive (SRA) of the National Center for Biotechnology Information (NCBI) database under project number PRJNA192205 and in the European Nucleotide Archive (ENA) database under accession id PRJEB9057.

## Additional information

**How to cite this article:** Vijay, N. *et al*. Evolution of heterogeneous genome differentiation across multiple contact zones in a crow species complex. *Nat. Commun.* **7,** 13195 doi: 10.1038/ncomms13195 (2016).

**Publisher's note:** Springer Nature remains neutral with regard to jurisdictional claims in published maps and institutional affiliations.

## Supplementary Material

Supplementary Information Supplementary Figures 1-8, Supplementary Tables 1-14, Supplementary Note 1 and Supplementary References
