# Supplementary material for: Evolution of heterogeneous genome differentiation across multiple contact zones in a crow species complex
Source: Nat Commun. 2016 Oct 31;7:13195. doi: 10.1038/ncomms13195 (PMC5095515; doi:10.1038/ncomms13195)
Supplement: Supplementary Information — Supplementary Figures 1-8, Supplementary Tables 1-14, Supplementary Note 1 and Supplementary References [file ncomms13195-s1.pdf]

Supplementary Figures

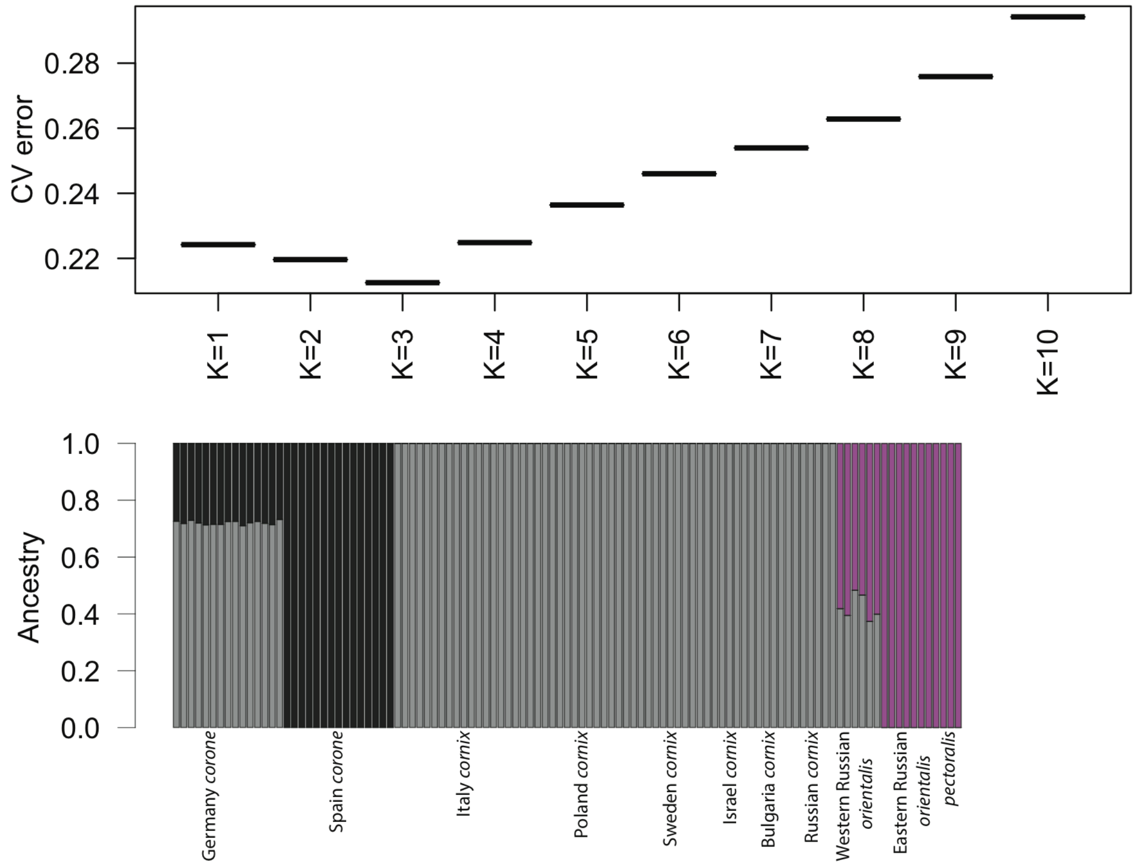

Supplementary Figure 1: Admixture analyses of the Eurasian crow species complex.

Cross validation analyses indicate that K=3 best describes population stratification (*top panel*). The genome-wide admixture plot of K=3 does not correspond to subspecies classification (*bottom panel*). Clear clusters emerge for the Spanish carrion (black), European hooded (grey) and the Eastern carrion and Chinese collared crow (pink). Both populations with black phenotypes adjacent to hybrid zones (German *corone* and Western Russian *orientalis*) are admixed.

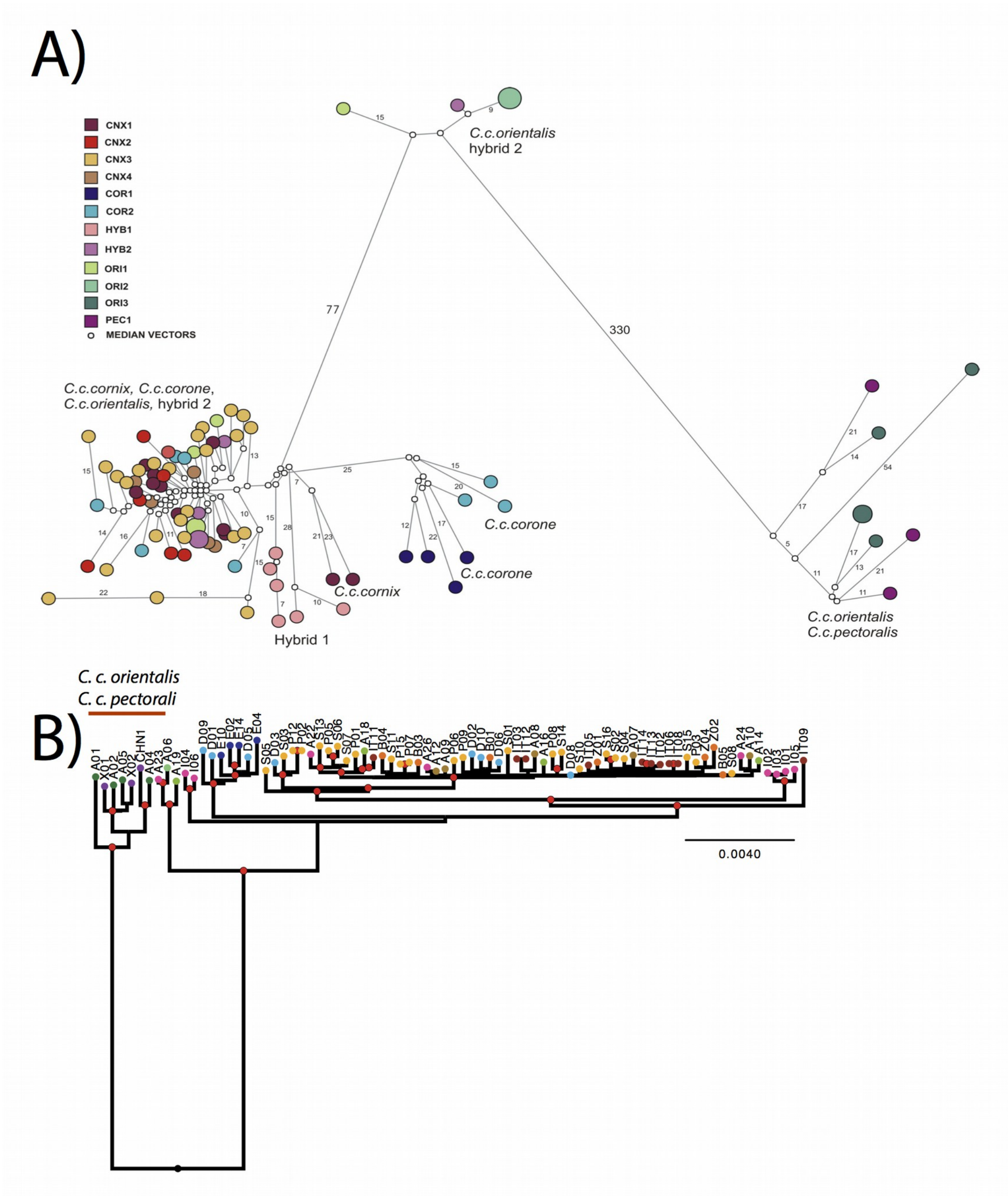

Supplementary Figure 2: Population structure as assessed by mitochondrial phylogenetic network analysis.

A) Mitochondrial median joining network reconstructed of the mitochondrial genome for haplotypes segregating across all populations. Number of mutations are indicated on the branches. Color of the nodes correspond to colors in Figure 1A.

B) Maximum likelihood-based phylogeny of 82 full mitochondrial genome sequences with the American crow used as the outgroup (pruned for image). Red nodes denote bootstrap support of 90% and greater. Tip color corresponds to abbreviated population names in panel A.

For population codes see Figure 1 of the main manuscript.

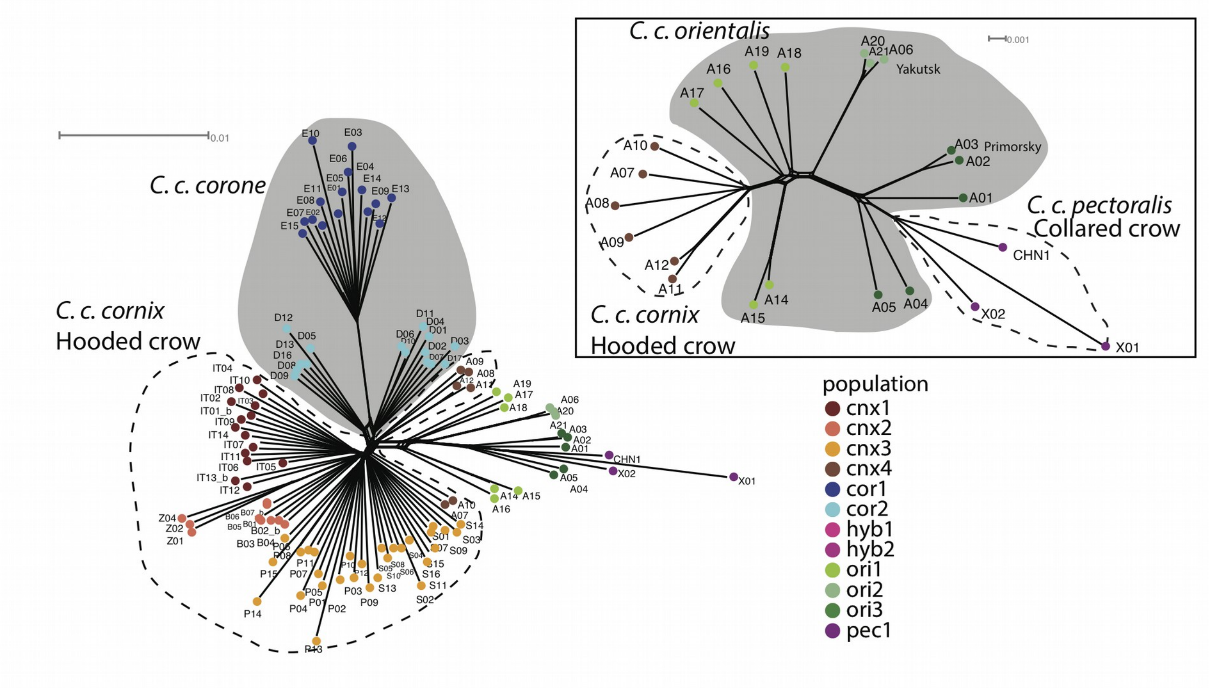

Supplementary Figure 3: Population structure as assessed by autosomal phylogenetic network analysis.

Splitstree network for all 107 individuals, excluding hybrids, based on autosomal SNPs. *Right*: The framed network separately depicts the relationships of the Eastern populations (cnx4, ori1, ori2, ori3, pec1).

The split network reconstruction demonstrates reticulate relationship of the German population (D individuals) between the entire hooded crow taxa and the Spanish carrion crow, consistent with population structure analyses in which the German individuals are admixed (Supplementary Figure 1). In addition, there is an ambiguous evolutionary relationship between the Russian *C. c. orientalis* adjacent to the Siberian hybrid zone and the eastern *C. c. orientalis* from Primorsky and Yakutsk, also supported by the mixed ancestry of these individuals in the admixture analyses. The figure further provides evidence that despite phenotypic similarity, *C. c. cornix* and *C. c. pectoralis* do not share the most recent common ancestor, but instead share most recent common ancestry with black populations.

For population codes see Figure 1 of the main manuscript.

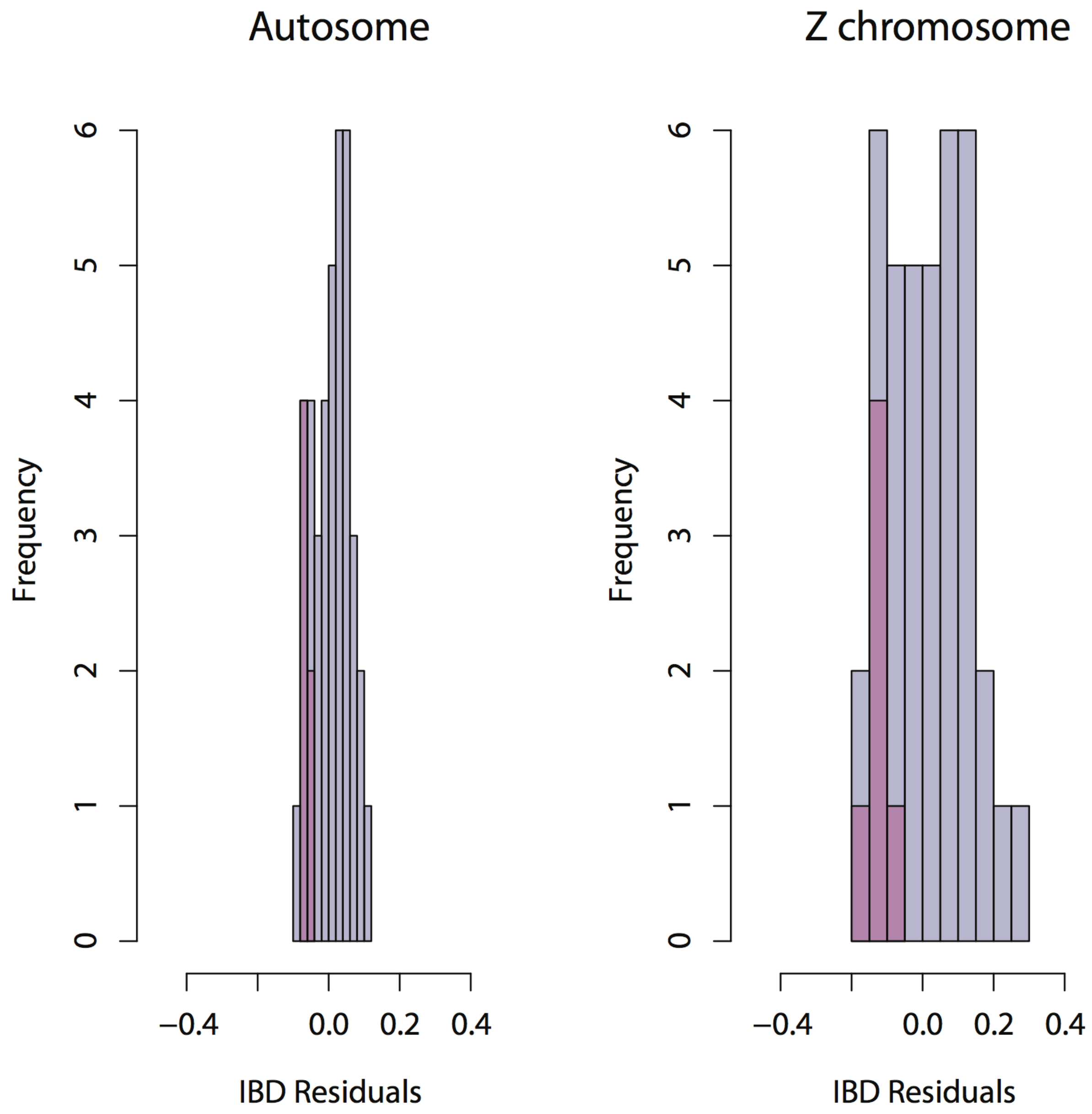

Supplementary Figure 4: Isolation by distance - residuals.

Between all populations under consideration, a correlation exists between genetic distance ($F_{ST}/(1-F_{ST})$) and geographic distance (isolation by distance). Residuals of the regression indicate how well genetic differentiation between a pair of populations is predicted by geographic distance. As expected, the residual distribution is centered around 0 with positive values indicating relative excess of genetic differentiation and negative values providing evidence for lower genetic differentiation than expected by geographic distance alone. All *C. c. cornix* population comparisons (pink) had extremely negative residuals relative to all other population comparisons (blue) for both autosomes and the sex chromosome.

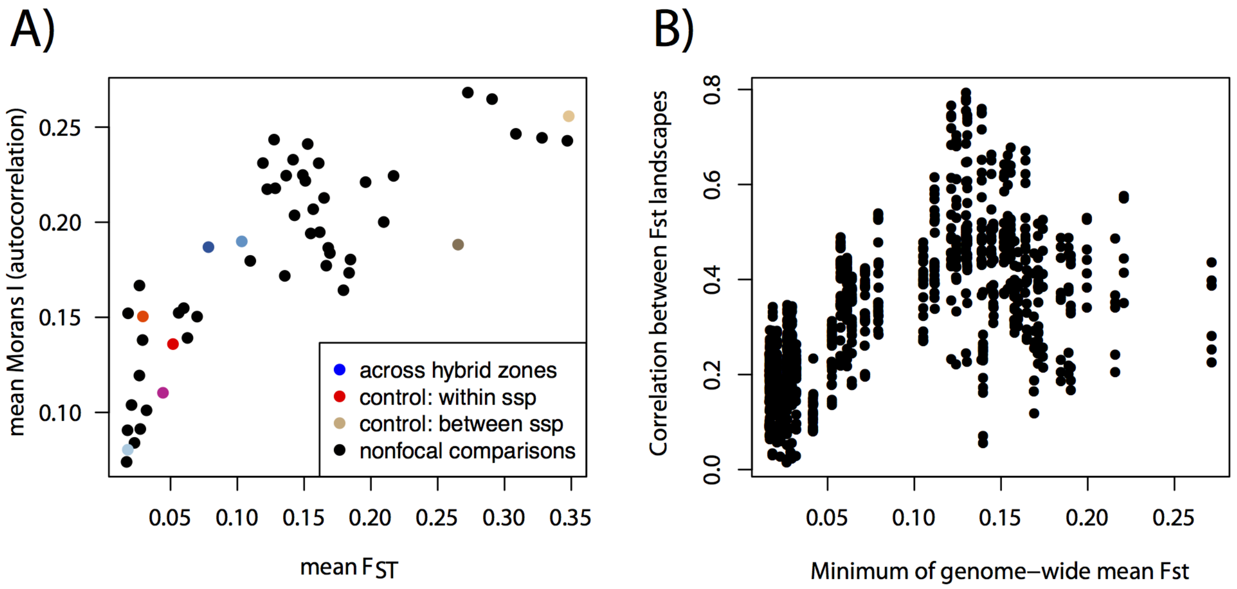

Supplementary Figure 5: Differentiation landscapes crystallize with increasing differentiation.

A) Relationship between mean genome-wide differentiation for each pair-wise population comparison and the level of autocorrelation of F_{ST} across the genome as measured by Moran's I and

B) Relationship between the minimum genome-wide differentiation of pairs from all possible population comparisons and similarity in the differentiation landscapes measured as the degree of correlation (Pearson's r).

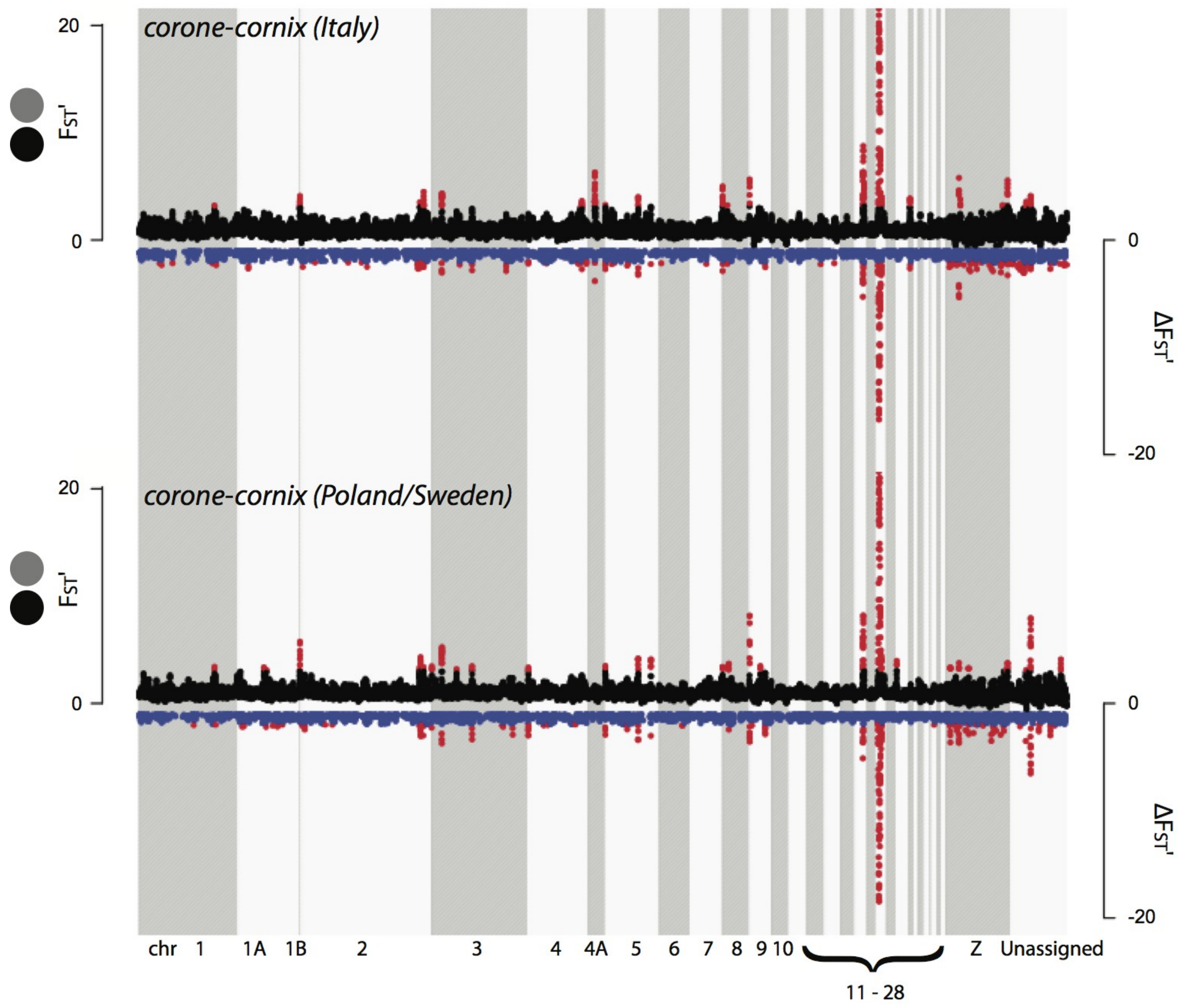

Supplementary Figure 6: Differentiation landscapes across the European hybrid zone.

Standardized genetic differentiation F_{ST}' (black, positive axis) and net differentiation $\Delta F_{ST}'$ (blue, mirrored to the negative axis) in 50 kb windows across the genome between Germany-Italy (=cor2-cnx1, above) vs. Germany-Poland/Sweden (=cor2-cnx3, below). Genomic regions of extreme differentiation (>99th percentile) are shown in in red for both F_{ST}' and $\Delta F_{ST}'$.

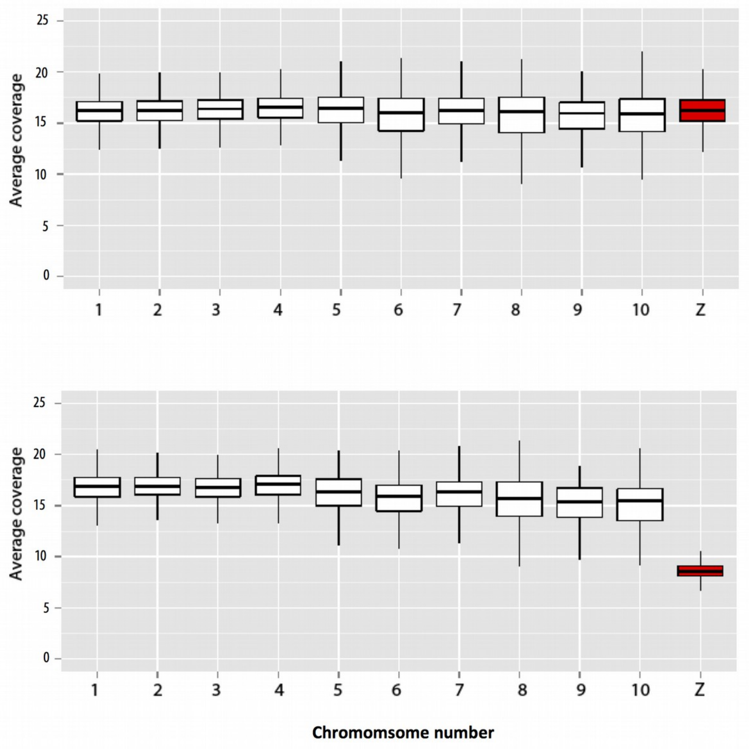

Supplementary Figure 7: Sexing by mean chromosome coverage.

Boxplots of mean coverage per 50kb window shown for a selection of autosomal chromosomes (white) with similar size and GC content as the sex chromosome (red). Males (*top*) are homogametic and show the same coverage for autosomes and the sex chromosome. Females (*bottom*) are the heterogametic sex and accordingly show half the coverage on the Z chromosome than on the autosomes.

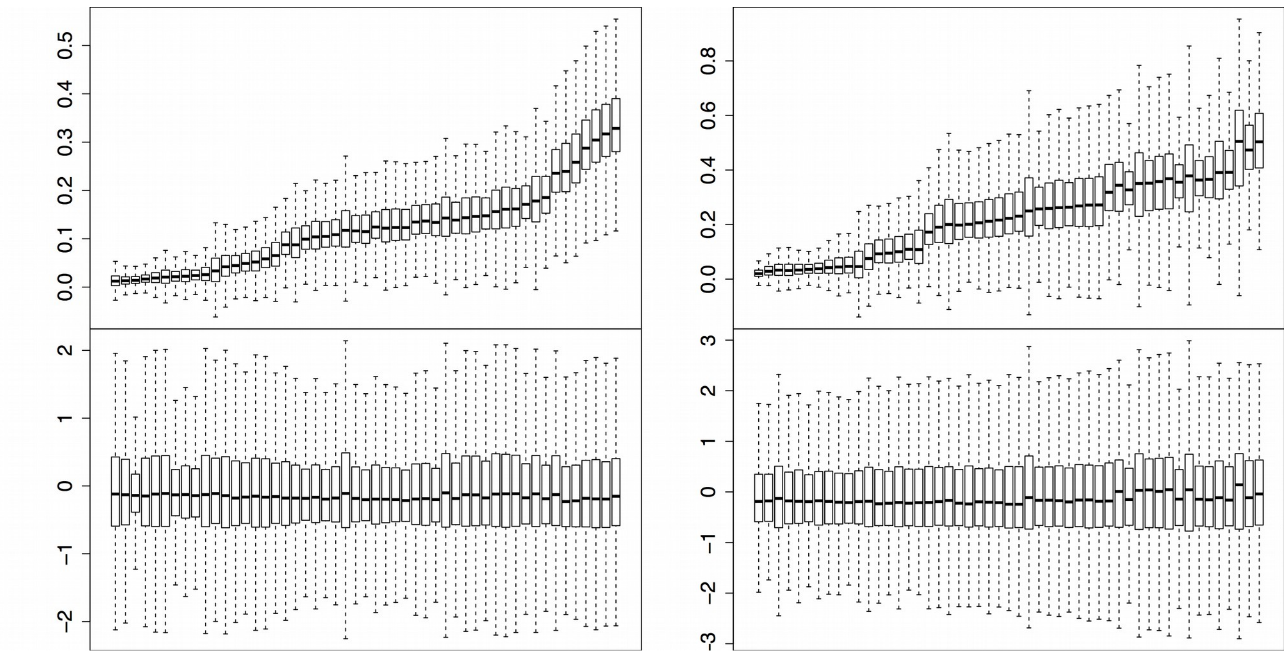

Supplementary Figure 8: Normalization of F_{ST} values.

F_{ST} (*upper row*) and z-transformed F_{ST}' (*lower row*) values for all possible pairwise comparisons shown separately for autosomes (*left*) and the Z-chromosome (*right*). The transformed values are centered around zero and show a similar, symmetric spread approximating a Normal Gaussian distribution. Straight horizontal lines depict the median, box margins indicate the interquartile range between 25 and 75% quantiles, whiskers extend to 1.5-times the interquartile range with values beyond shown as points.

Supplementary Tables

Supplementary Table 1. Genome-wide estimates of mean F_{ST} based on genotype calls from GATK. Upper triangle refers to values from the autosome and lower triangle refers to values from the Z chromosome.

<table>
<tr><th>GroupID</th><th>bra1</th><th>cnx1</th><th>cnx2</th><th>cnx3</th><th>cnx4</th><th>cor1</th><th>cor2</th><th>ori1</th><th>ori2</th><th>ori3</th><th>pec1</th></tr>
<tr><td>bra1</td><td></td><td colspan="4">0.26</td><td>0.34</td><td>0.28</td><td>0.32</td><td>0.47</td><td>0.31</td><td>0.35</td></tr>
<tr><td>cnx1</td><td rowspan="4">0.39</td><td></td><td>0.02</td><td>0.02</td><td>0.03</td><td>0.15</td><td>0.03</td><td>0.07</td><td>0.17</td><td>0.13</td><td>0.15</td></tr>
<tr><td>cnx2</td><td>0.04</td><td></td><td>0.02</td><td>0.02</td><td>0.15</td><td>0.03</td><td>0.06</td><td>0.17</td><td>0.12</td><td>0.14</td></tr>
<tr><td>cnx3</td><td>0.04</td><td>0.03</td><td></td><td>0.01</td><td>0.14</td><td>0.02</td><td>0.05</td><td>0.15</td><td>0.12</td><td>0.14</td></tr>
<tr><td>cnx4</td><td>0.06</td><td>0.06</td><td>0.04</td><td></td><td>0.15</td><td>0.03</td><td>0.05</td><td>0.17</td><td>0.12</td><td>0.14</td></tr>
<tr><td>cor1</td><td>0.49</td><td>0.26</td><td>0.25</td><td>0.23</td><td>0.29</td><td></td><td>0.1</td><td>0.16</td><td>0.25</td><td>0.18</td><td>0.2</td></tr>
<tr><td>cor2</td><td>0.41</td><td>0.04</td><td>0.04</td><td>0.02</td><td>0.05</td><td>0.19</td><td></td><td>0.06</td><td>0.16</td><td>0.11</td><td>0.13</td></tr>
<tr><td>ori1</td><td>0.38</td><td>0.13</td><td>0.12</td><td>0.11</td><td>0.09</td><td>0.28</td><td>0.11</td><td></td><td>0.12</td><td>0.07</td><td>0.1</td></tr>
<tr><td>ori2</td><td>0.52</td><td>0.36</td><td>0.36</td><td>0.34</td><td>0.37</td><td>0.51</td><td>0.35</td><td>0.27</td><td></td><td>0.15</td><td>0.19</td></tr>
<tr><td>ori3</td><td>0.34</td><td>0.24</td><td>0.23</td><td>0.24</td><td>0.21</td><td>0.34</td><td>0.22</td><td>0.13</td><td>0.35</td><td></td><td>0.04</td></tr>
<tr><td>pec1</td><td>0.37</td><td>0.29</td><td>0.29</td><td>0.3</td><td>0.29</td><td>0.41</td><td>0.28</td><td>0.22</td><td>0.48</td><td>0.06</td><td></td></tr>
</table>

Supplementary Table 2. Sample information and population genetic summary statistics. Taxon delineation (following sub-species classification see above), population (country, region of provenance), geographic location (latitude and longitude in decimal degrees WGS84), number of sampled individuals (N: mitochondrial, autosomal data), identifier of population groups as by **Figure 1 (**main manuscript), sample provider (see below table), the genome-wide average (median) for a series of population genetic summaries and linkage disequilibrium (LD).

	**Taxon**	**Population**	**Lat**	**Lon**	**N**	**Group ID**	**Provider**	**Watterson's θ**	**π**	**Tajima's D**	**Fay's H**	**Fu & Li's D**	**LD[r^2]**
Corvus corone corone/cornix/pallescens/orientalis/pectoralis complex	*Corvus (c.) corone*	Spain	42.50	-5.48	15, 4	cor1	1	0.0012	0.0012	-0.149	0.364	0.258	NA
		Germany	47.71	9.12	15, 8	cor2	1	0.0019	0.0013	-1.066	0.372	-1.278	0.121
	Corvus (c.) cornix	Poland	52.23	21.01	15, 10	cnx3	1	0.0019	0.0013	-1.116	0.286	-1.371	NA
		Sweden Rimbo	59.80	18.37	5, 5		1						
		Sweden Uppsala	59.89	17.64	10, 9		1						
		Italy	41.73	12.27	14, 11	cnx1	2	0.0032	0.0017	-1.82	0.308	-3.859	0.130
	Corvus (c.) pallescens	Bulgaria Sofia	42.70	23.33	4, 4	cnx2	3	0.0042	0.0022	-1.974	0.369	-3.961	NA
		Bulgaria Stora Zagora	42.42	25.63	3, 0		3						
		Israel*	32.07	34.74	3, 3		4						
	Corvus (c.) cornix	Russia Kirov	58.60	49.67	3, 3	cnx4	5	0.0015	0.0013	-0.651	0.525	-0.940	0.159
		Russia Tyumen	56.27	70.45	1, 0		5						
		Russia Novosibirsk	54.85	83.10	2, 2		5						
	Corvus (c.) orientalis	Russia Krasnoyarsky	55.96	92.88	4, 4	ori1	5	0.0017	0.0013	-0.829	0.546	-1.277	0.127
		Russia Tuva	50.11	96.10	2, 1		5						
		Russia Yakutsk	62.02	129.72	3, 3	ori2	5	0.0018	0.0014	-0.902	0.597	-1.311	NA
		Russia	43.35	132.20	5, 5	ori3	5	0.0009	0.0010	0.590	0.295	0.668	NA

	Taxon	Population	Lat	Lon	N	Group ID	Provider	Watterson's θ	π	Tajima's D	Fay's H	Fu & Li's D	LD[r^2]
		Primorsky											
	C. (c.) corone x *cornix*	Ireland	54.31	-5.63	6, 6	hyb1	6	0.0013	0.0012	-0.269	0.487	-0.362	0.144
	C. (c.) orientalis x *cornix*	Russia Kemerovo	56.07	89.03	5, 5	hyb2	5	0.0016	0.0014	-0.587	0.545	-0.953	0.147
	C. (c.) pectoralis	Guangxi	22.76	108.31	1, 1	pec1	7	0.0041	0.0033	-1.187	1.065	-2.620	NA
		-	-	-	2, 2		8,9						
Outgroups	*C. brachyrhynchos*	**-	-	-	6, 0	bra1	1,10	NA	NA	NA	NA	NA	NA
	C. frugilegus	-	-	-	2, 0	-	1	NA	NA	NA	NA	NA	NA
	C. monedula	-	-	-	2, 0	-	1	NA	NA	NA	NA	NA	NA

Sample provider: 1. as published in Poelstra et al. 2014, 2. Centro Recupero Fauna Selvatica LIPU di Roma , Rome, Italy; 3. Wildlife rehabilitation and breeding centre-Green Balkans, Stara Zagora, Bulgaria with aid from Nayden Chakarov; 4. Tel Aviv University, Zoological Museum, Israel, serial numbers: Av.16801/16889/16756; 5. Alexey Kryukov, Institute of Biology and Soil Science, Vladivostok, Russia; 6. Jochen Wolf, Uppsala University, Sweden in co-operation with Jamie Dick from Queen's University, Belfast, Ireland and Chris Harrod Queen Mary University London, U.K.; 7. Lei Fumin, Chinese Academy of Sciences, Beijing, China; 8. American Museum of Natural History, museum ID AMNH 261595; 9. Swedish Museum of National History, museum ID NRM 570709; 10. Genome-wide re-sequencing data from one individual was downloaded from the NCBI short read archive (SRA) – accession numbers SRX329030 and SRX329031 [1].

* original sample numbers: TAU 16801, 16756, 16889, 17013

Supplementary Table 3. ABBA-BABA test statistics based on the phylogeny ((H1:H2)H3)H4). H1, H2 and H3 represent relationships between 3 crow populations with the outgroup H4 shared by all. A positive value of the D-statistic reflects higher gene flow between H2 and H3 than with H1 and a negative value means reflects higher gene flow between H1 and H3 than with H2 (see Supplementary Figure 5 in [2]). Based on the significance of the test, populations with evidence for gene flow are highlighted in bold. The D-statistic was estimated in *ANGSD*. *Abbreviations:* ABBA/BABA: number of ABBA/BABA sites, D: Patterson's D statistic. The standard error (SE) of the D statistic is rounded to 2 decimal places. Population comparisons informative about gene flow across hybrid zones are highlighted with a grey background. A Z-score value of 3 and above is considered significant**.**

**contact zones**	**Populations**			**ABBA-BABA test**				
	**H1**	**H2**	**H3**	**ABBA**	**BABA**	**D**	**SE**	**Z**
**Europe**	**Cor2**	Cor1	**Cnx1**	86282	136678	-0.226	0.0028	**-80.7575**
	**Cnx1**	Cor1	**Cor2**	110690	136678	-0.1051	0.0030	**-34.9111**
	Cnx1	**Cor2**	**Cor1**	110690	86282	0.1239	0.0030	**41.9398**
**Siberia**	Ori3	**Cnx4**	**Ori1**	140071	94104	0.1963	0.0030	**64.5971**
	**Ori1**	Ori2	**Cnx4**	97420	115996	-0.0870	0.0031	**-28.4764**
	**Ori1**	Ori3	**Cnx4**	74503	140071	-0.3056	0.0029	**-106.1954**
	**Ori2**	Cnx4	**Ori1**	115996	122767	-0.0284	0.0031	**-9.1730**
	**Ori2**	Cnx4	**Ori3**	77066	102838	-0.1433	0.0031	**-46.8227**
	**Ori1**	Ori3	**Ori2**	94569	141119	-0.1975	0.0031	**-64.5716**
	**Ori1**	Cnx4	**Ori2**	97420	122767	-0.1151	0.0031	**-37.4883**
	Ori3	**Cnx4**	**Ori2**	124120	102838	0.0938	0.0032	**29.7128**
	Ori1	**Ori2**	**Ori3**	94569	88410	0.0337	0.0031	**10.8728**
	**Ori1**	Cnx4	**Ori3**	74503	94104	-0.1163	0.0031	**-38.0447**
	Ori3	**Ori2**	**Cnx4**	124120	77066	0.2339	0.0030	**77.0083**
**East Asia**	Ori1	**Ori3**	**Pec1**	108805	82371	0.1383	0.0031	**44.1084**
	**Ori3**	Ori2	**Pec1**	88059	105516	-0.0902	0.0031	**-28.7712**
	**Pec1**	Ori1	**Ori3**	106693	108805	-0.0098	0.0032	**-3.0640**
	Pec1	**Ori2**	**Ori3**	109945	105516	0.0206	0.0032	**6.4845**
	Pec1	**Ori2**	**Ori1**	151629	74719	0.3398	0.0029	**118.4002**
	Ori3	**Ori2**	**Ori1**	140627	87940	0.2305	0.0030	**75.7677**
	Ori1	**Ori2**	**Pec1**	83697	74719	0.0567	0.0032	**17.7938**
	Pec1	**Ori3**	**Ori1**	106693	82371	0.1286	0.0031	**41.0296**
	Ori1	**Ori2**	**Ori3**	94502	87940	0.0360	0.0031	**11.5416**
	Pec1	**Ori1**	**Ori2**	151629	83697	0.2887	0.0030	**96.2869**
	Pec1	**Ori3**	**Ori2**	109945	88059	0.1105	0.0031	**35.4039**
	**Ori1**	Ori3	**Ori2**	94502	140627	-0.1962	0.0031	**-63.8719**
	Ori3	**Ori2**	**Ori1**	141119	88410	0.2296	0.0030	**75.5343**

In the European hybrid zone, the D-statistic supported gene flow between Cor2 and Cnx1

across the hybrid zone regardless of the choice of tree, ((Cor2:Cor1)Cnx1)Outgroup or ((Cnx1:Cor1)Cor2)O. Similarly, in the Siberian hybrid zone gene flow between Cnx4 and Ori1 across the hybrid zone was supported by all trees ((Ori3:Cnx4)Ori1)O, ((Ori1:Ori3)Cnx4)O and ((Ori1:Ori2)Cnx4)O. In the zone of contact between Pec1 and Ori3, gene flow between Pec1 and Ori3 was supported by the trees ((Ori1:Ori3)Pec1)O, ((Ori3:Ori2)Pec1)O and ((Pec1:Ori1)Ori3)O.

Supplementary Table 4. Distribution of correlation estimates (corr) of central summary statistics supporting linked selection shared among populations as a central element shaping heterogeneous genomic differentiation. Subscripts *i, j* symbolize all possible combinations between two populations $i=1...n$ and $j=i+1....n$ for within-populations measures*;* Capital letters *I, J* symbolize inter-population statistics. Correlations were conducted between of all possible population comparisons I and J excluding comparisons with pseudo-replicated populations (e.g. I=ori1,cnx1, J=ori1,pec).

summary statistic	Min.	Lower 5%	Mean	Median	Max.	Nr. comparisons
corr($\rho_{i,}$, ρ_j)	0.0664	0.06775	0.3700	0.4119	0.8052	15
corr($LD_{i,}$, LD_j)	0.215	0.3336	0.561	0.5841	0.8775	45
corr(π_i, π_j)	0.6117	0.6522	0.8166	0.8381	0.9821	45
corr($\rho_{i,}$, π_i)	-0.0022	-0.0022	0.1303	0.1157	0.3710	10
corr(LD_i, π_i)	-0.1066	-0.1533	-0.2721	-0.2947	-0.3716	10
corr(π_i, μ)	0.0749	0.0886	0.1451	0.1257	0.2488	10
corr(π_i, GD_i)	0.0565	0.0395	-0.0415	-0.0664	-0.0859	10
corr($\pi_{i\ or\ j}$, $F_{ST\ I=i,j}$)	-0.0337	-0.0708	-0.218	-0.2086	-0.5544	90
corr(π_i, PBS_i)	-0.5661	-0.46	-0.2528	-0.2406	-0.1132	10
corr($\rho_{i\ or\ j}$, $F_{ST\ I=i,j}$)	0.0556	0.0178	-0.1696	-0.1847	-0.2985	90
corr(ρ_i, PBS_i)	0.0268	-0.0460	-0.1770	-0.1880	-0.3010	10
corr($LD_{i\ or\ j}$, $F_{ST\ I=i,j}$)	0.0856	0.1307	0.3555	0.3848	0.6027	90
corr(LD_i, PBS_i)	0.1832	0.192	0.3665	0.3877	0.5365	10
corr($F_{ST\ I}$, $F_{ST\ J}$)	0.0161	0.0962	0.324	0.3113	0.7935	900
corr(PBS_i, PBS_j)	0.2307	0.2401	0.4853	0.3956	0.9866	45
corr($F_{ST\ I}$, $D_{xy\ I}$)	-0.2284	-0.2164	-0.1409	-0.1457	-0.02	45

Supplementary Table 5. Testing for the relationship between $\pi \sim$ GD(gene density) + ρ + GD* ρ using multiple regression analyses. Background selection predicts a negative relationship between π and the density of potential targets for selection here approximated by gene density at low recombination rate (here approximated by ρ [3]. Statistically this corresponds to negative slope estimates for gene density, positive slopes for recombination rate and, importantly, a positive interaction term. To normalize model residuals π and ρ were transformed with the natural logarithm, gene density with the square root.

Population	intercept	sqrt(GD)	log(ρ)	interaction	R^2
cor1	-6.68***	-0.23***	0.08***	0.13***	0.05
cor2	-6.62**	-0.34***	0.06***	0.12***	0.06
cnx1	-6.35***	-0.15***	0.09***	0.06***	0.10
cnx2	-6.14***	-0.09***	0.09***	0.05**	0.16
cnx3	-6.64***	-0.29***	0.07***	0.07***	0.06
cnx4	-6.61***	-0.22***	0.03***	0.02	0.03
ori1	-6.56***	-0.21***	0.03***	0.04**	0.05
ori2	-6.50***	-0.15***	0.00*	0.01**	0.02
ori3	-6.87***	-0.12***	0.04***	-0.02	0.02
pec1	-5.63***	0.14***	0.03***	0.05***	0.06

Supplementary Table 6. Comparison of population statistics of 'contact zone peaks' with genome-wide background estimates. Statistically significant (α=0.05) comparisons are highlighted with a grey background. n. s.= not significant.

	corone-cornix **contact zone**		***cornix-orientalis*** **contact zone**		***orientalis-pectoralis*** **contact zone**	
	**Direction**	**p-value**	**Direction**	**p-value**	**Direction**	**p-value**
**Population statistics**						
**D_{xy}**	Lower	1.93E-07	n. s.	7.80E-01	n. s.	1.57E-01
**π: black**	Lower	< 2.20E-16	Lower	6.59E-11	Lower	1.05E-06
**π: pied**	Lower	1.52E-11	Lower	8.59E-06	Lower	2.37E-12
**FayWu's H: black**	Lower	< 2.20E-16	Lower	1.52E-14	n. s.	1.26E-01
**FayWu's H: pied**	Lower	1.18E-15	Lower	2.78E-05	Lower	9.24E-05
**LD (r^2): black**	Higher	< 2.20E-16	Higher	1.13E-13	Higher	2.47E-15
**LD (r^2): pied**	Higher	2.76E-14	Higher	3.89E-11	Higher	1.54E-15
**PBS: black**	Higher	< 2.20E-16	n. s.	3.49E-01	Higher	< 2.20E-16
**PBS: pied**	Higher	< 2.20E-16	Higher	< 2.20E-16	Higher	< 2.20E-16
**Haplotype statistics**						
**iHHa/iHHd: black**	Longer	< 2.20E-16/ <2.20E-16	Longer	7.56E-15/ 2.55E-08	Longer	<2.20E-16/ 8.34E-11
**iHHa/iHHd: pied**	Longer	< 2.20E-16/ < 2.20E-16	Longer	8.74E-15/ 2.73E-07	Longer	<2.20E-16/ < 2.20E-16
**abs(iHS): black**	Higher	7.41E-06	Higher	1.73E-07	Higher	1.45E-05
**abs(iHS): pied**	n. s.	1.70E-01	Higher	2.76E-04	Higher	6.74E-08
**abs(nSL): black**	n. s.	9.94E-01	Higher	2.40E-11	Higher	1.53E-10
**abs(nSL): pied**	n. s.	1.26E-01	Higher	3.73E-07	Higher	4.23E-13
**XP-EHH**	Higher	1.24E-05	n. s.	3.75E-01	Lower	4.08E-02

Supplementary Table 7. Comparison of population statistics of contact zone peaks with non-overlapping windows from 'shared peaks'. Statistically significant (α=0.05) comparisons are highlighted with a grey background. n. s.= not significant.

	***corone-cornix* contact zone**		***cornix-orientalis* contact zone**		***orientalis-pectoralis* contact zone**	
	**Direction**	**p-value**	**Direction**	**p-value**	**Direction**	**p-value**
**Population statistics**						
**D_{xy}**	n. s.	3.01E-01	n. s.	6.23E-02	n. s.	2.04E-01
**π: black**	Lower	1.73E-03	n. s.	1.64E-01	Lower	1.96E-02
**π: pied**	n. s.	8.62E-01	n. s.	1.47E-01	Lower	3.28E-02
**FayWu's H: black**	Lower	1.35E-03	n. s.	2.50E-01	n. s.	5.88E-01
**FayWu's H: pied**	Higher	2.55E-04	n. s.	8.69E-01	Higher	1.31E-02
**LD (r^2): black**	n. s.	3.66E-01	n. s.	9.22E-01	n. s.	1.03E-01
**LD (r^2): pied**	Higher	2.29E-02	n. s.	3.20E-01	n. s.	7.23E-01
**PBS: black**	Higher	2.58E-02	Higher	7.55E-03	Higher	8.30E-09
**PBS: pied**	n. s.	8.91E-02	n. s.	4.99E-01	Higher	2.89E-08
**Haplotype statistics**						
**iHHa/iHHd: black**	n. s. / Longer	8.95E-02/ 3.74E-02	n. s.	7.70E-01/ 1.90E-01	n. s.	1.20E-01/ 7.84E-01
**iHHa/iHHd: pied**	n. s.	5.69E-01/ 1.09E-01	n. s.	3.78E-02/ 9.13E-01	Longer	2.75E-03/ 8.65E-03
**abs(iHS): black**	n. s.	6.46E-01	Higher	7.79E-04	n. s.	8.53E-01
**abs(iHS): pied**	n. s.	6.50E-01	n. s.	6.39E-01	n. s.	1.57E-01
**abs(nSL): black**	n. s.	1.22E-01	Higher	3.47E-02	Higher	2.77E-02
**abs(nSL): pied**	Lower	6.82E-03	n. s.	2.28E-01	Higher	4.39E-03
**XP-EHH**	Higher	9.95E-10	n. s.	1.63E-01	Lower	1.16E-03

Supplementary Table 8. List of genes located within remaining 'contact zone' peaks (99th percentile ΔF_{ST}') and divergent cacti.

		corone-cornix		*cornix-orientalis*		*orientalis-pectoralis*	
scaffold	gene	outlier peak	divergent cactus	outlier peak	divergent cactus	outlier peak	divergent cactus
scaffold_32	ABCA1	0	1	0	0	0	0
scaffold_78	ABCA5	1	0	0	0	0	0
scaffold_21	ADAMTS12	0	1	0	0	0	0
scaffold_60	ADAP2	1	0	0	0	0	0
scaffold_108	ADARB1	1	0	0	0	0	0
scaffold_4	ADD1	0	1	0	0	0	0
scaffold_29	ADORA2A	1	0	0	0	0	0
scaffold_4	AFAP1	0	1	0	0	0	0
scaffold_29	ANHX	1	0	0	0	0	0
scaffold_60	ANKFN1	1	1	0	0	0	0
scaffold_13	APBA2	0	0	0	0	1	0
scaffold_78	APOH	1	1	0	0	0	0
scaffold_23	APP	0	0	0	1	1	0
scaffold_0	AREL1	0	0	1	0	0	0
scaffold_104	ARFIP2	0	0	1	0	0	0
scaffold_78	ARSG	1	0	0	0	0	0
scaffold_60	ATAD5	1	0	0	0	0	0
scaffold_148	ATP6V1F	0	0	0	1	1	0
scaffold_100	ATPIF1	0	0	0	1	1	0
scaffold_78	AXIN2	1	1	0	0	0	0
scaffold_29	BCR	1	1	0	0	0	0
scaffold_78	BPTF	1	1	0	0	0	0
scaffold_80	C1D	0	0	1	0	0	0
scaffold_48	C2CD5	0	0	0	0	0	1
scaffold_184	C3	0	1	0	0	0	0
scaffold_60	CA10	1	1	0	0	0	0
scaffold_29	CABP1	0	0	0	1	0	0
scaffold_12	CACNA1D	0	1	0	0	0	0
scaffold_78	CACNG1	1	1	0	0	0	1
scaffold_78	CACNG4	1	1	0	0	0	1
scaffold_78	CACNG5	1	0	0	0	0	1
scaffold_148	CALU	0	0	0	1	1	0
scaffold_0	CAPN3	0	0	0	0	1	0
scaffold_3	CCDC126	0	0	1	0	0	0
scaffold_21	CCDC152	0	1	0	0	0	0

scaffold_78	CEP112	1	1	0	0	0	0
scaffold_60	CEP95	1	1	0	0	0	0
scaffold_100	CITED4	0	0	0	1	1	0
scaffold_55	CLCN6	0	0	1	0	0	0
scaffold_60	COIL	1	0	0	0	0	0
scaffold_60	COPRS	1	0	0	0	0	0
scaffold_0	CPT1A	0	0	0	0	1	0
scaffold_29	CRKL	1	0	0	0	0	0
scaffold_60	CRLF3	1	0	0	0	0	0
scaffold_5	CSMD3	0	0	1	0	0	0
scaffold_100	CTPS1	0	0	0	0	1	0
scaffold_31	CXCL14	0	1	0	0	0	0
scaffold_21	DCAF12	0	1	0	0	0	0
scaffold_104	DCHS1	0	0	1	0	0	0
scaffold_60	DDX5	1	0	0	0	0	0
scaffold_29	DEPDC5	1	0	0	0	0	0
scaffold_60	DGKE	1	0	0	0	0	0
scaffold_74	DHFR	0	0	0	0	1	0
scaffold_2	DMD	0	1	0	0	0	0
scaffold_100	DNAJC8	0	0	0	1	1	0
scaffold_67	DOCK1	0	1	0	0	0	0
scaffold_0	EGLN3	0	1	0	0	0	0
scaffold_44	ERCC6L	1	0	0	0	0	0
scaffold_60	ERN1	1	1	0	0	0	0
scaffold_48	ETNK1	0	0	0	0	0	1
scaffold_100	EYA3	0	0	0	1	1	0
scaffold_5	FAM135B	0	0	1	0	0	0
scaffold_16	FAM172A	0	1	0	0	0	0
scaffold_13	FAM189A1	0	0	0	0	1	0
scaffold_108	FAM207A	1	0	0	0	0	0
scaffold_78	FAM20A	1	1	0	0	0	0
scaffold_13	FAN1	0	0	0	0	1	0
scaffold_96	FANCF	0	0	0	0	1	0
scaffold_55	FBXO2	0	0	1	0	0	0
scaffold_72	FGD3	0	0	0	0	0	1
scaffold_86	FRAS1	0	1	0	0	0	0
scaffold_54	FSD1L	0	1	0	0	0	0
scaffold_9	FSTL5	0	1	0	0	0	0
scaffold_0	GANC	0	0	0	0	1	0
scaffold_96	GAS2	0	0	0	0	1	0
scaffold_29	GGT1	1	0	0	0	0	0

scaffold_64	GJA9	0	0	0	1	1	0
scaffold_78	GNA13	1	1	0	0	0	0
scaffold_29	GNAZ	1	0	0	0	0	0
scaffold_148	GOLGB1	0	0	0	1	1	0
scaffold_41	GPC4	0	1	0	0	0	0
scaffold_16	GPR98	0	1	0	0	0	0
scaffold_29	GUCD1	1	1	0	0	0	0
scaffold_8	GUCY2C	1	0	0	0	0	0
scaffold_78	HELZ	1	1	0	0	0	1
scaffold_10	HIF1AN	0	1	0	0	0	0
scaffold_60	HLF	1	1	0	0	0	0
scaffold_100	HNRNPR	0	0	0	1	1	0
scaffold_41	HPRT1	0	1	0	0	0	0
scaffold_100	HTR1D	0	0	0	1	1	0
scaffold_104	ILK	0	0	1	0	0	0
scaffold_67	INPP5A	0	0	0	1	1	0
scaffold_100	KDM1A	0	0	0	1	1	0
scaffold_32	KIAA1024L	0	1	0	0	0	0
scaffold_55	KIAA2013	0	0	0	0	0	1
scaffold_21	KIF24	0	1	0	0	0	0
scaffold_29	KLHL22	1	0	0	0	0	0
scaffold_60	KPNA2	1	0	0	0	0	0
scaffold_58	LAMC1	0	0	1	0	0	0
scaffold_58	LAMC2	0	0	1	0	0	0
scaffold_4	LCORL	0	0	1	0	0	0
scaffold_9	LEF1	0	1	0	0	0	0
scaffold_0	LOC104684395	0	0	1	0	0	0
scaffold_0	LOC104684412	0	0	1	0	0	0
scaffold_147	LOC104684608	0	0	0	0	1	0
scaffold_148	LOC104684611	0	0	0	1	1	0
scaffold_148	LOC104684612	0	0	0	1	1	0
scaffold_148	LOC104684614	0	0	0	1	1	0
scaffold_148	LOC104684617	0	0	0	1	1	0
scaffold_148	LOC104684620	0	0	0	1	1	0
scaffold_16	LOC104685227	0	0	1	0	0	0
scaffold_16	LOC104685285	0	0	1	0	0	0
scaffold_172	LOC104685527	0	0	0	0	0	1
scaffold_172	LOC104685528	0	0	0	0	0	1
scaffold_172	LOC104685529	0	1	0	0	0	1
scaffold_0	LOC104686721	0	1	0	0	0	0
scaffold_23	LOC104686989	0	0	0	0	1	0

scaffold_23	LOC104686990	0	0	0	0	1	0
scaffold_29	LOC104688547	0	0	0	1	0	0
scaffold_29	LOC104688548	0	0	0	1	0	0
scaffold_41	LOC104691252	0	1	0	0	0	0
scaffold_41	LOC104691256	0	1	0	0	0	0
scaffold_44	LOC104691586	1	0	0	0	0	0
scaffold_0	LOC104692534	0	0	1	1	1	0
scaffold_54	LOC104693197	0	1	0	0	0	0
scaffold_55	LOC104693238	0	0	1	0	0	0
scaffold_55	LOC104693240	0	0	1	0	0	0
scaffold_55	LOC104693243	0	0	1	0	0	1
scaffold_55	LOC104693244	0	0	1	0	0	1
scaffold_55	LOC104693356	0	0	1	0	0	0
scaffold_55	LOC104693357	0	0	1	0	0	0
scaffold_55	LOC104693358	0	0	1	0	0	0
scaffold_55	LOC104693359	0	0	1	0	0	0
scaffold_55	LOC104693361	0	0	0	0	0	1
scaffold_56	LOC104693462	0	1	0	0	0	0
scaffold_57	LOC104693535	0	1	0	0	0	0
scaffold_60	LOC104694234	1	0	0	0	0	0
scaffold_60	LOC104694245	1	1	0	0	0	0
scaffold_60	LOC104694249	1	0	0	0	0	0
scaffold_60	LOC104694259	1	0	0	0	0	0
scaffold_60	LOC104694264	1	0	0	0	0	0
scaffold_60	LOC104694265	1	0	0	0	0	0
scaffold_60	LOC104694356	1	0	0	0	0	0
scaffold_64	LOC104694723	0	0	0	1	1	0
scaffold_64	LOC104694803	0	0	0	1	1	0
scaffold_668	LOC104694920	0	0	1	0	0	0
scaffold_67	LOC104694926	0	0	0	0	1	0
scaffold_7	LOC104695390	0	1	0	0	0	0
scaffold_74	LOC104695830	0	0	0	1	1	0
scaffold_78	LOC104696000	1	0	0	0	0	0
scaffold_78	LOC104696001	1	0	0	0	0	0
scaffold_78	LOC104696015	1	0	0	0	0	0
scaffold_78	LOC104696026	1	0	0	0	0	0
scaffold_9	LOC104697080	0	0	0	0	0	1
scaffold_9	LOC104697081	0	0	0	0	0	1
scaffold_9	LOC104697082	0	0	0	0	0	1
scaffold_9	LOC104697083	0	0	0	0	0	1
scaffold_9	LOC104697098	0	1	0	0	0	0

scaffold_9	LOC104697283	0	0	0	0	0	1
scaffold_957	LOC104697675	0	0	0	0	1	0
scaffold_104	LOC104698069	0	0	1	0	0	0
scaffold_104	LOC104698070	0	0	1	0	0	0
scaffold_104	LOC104698072	0	0	1	0	0	0
scaffold_104	LOC104698073	0	0	1	0	0	0
scaffold_104	LOC104698074	0	0	1	0	0	0
scaffold_104	LOC104698075	0	0	1	0	0	0
scaffold_104	LOC104698076	0	0	1	0	0	0
scaffold_104	LOC104698077	0	0	1	0	0	0
scaffold_104	LOC104698078	0	0	1	0	0	0
scaffold_104	LOC104698080	0	0	1	0	0	0
scaffold_104	LOC104698081	0	0	1	0	0	0
scaffold_104	LOC104698102	0	0	1	0	0	0
scaffold_104	LOC104698103	0	0	1	0	0	0
scaffold_104	LOC104698105	0	0	1	0	0	0
scaffold_0	LOC104698623	0	1	0	0	0	0
scaffold_34	LRCH1	0	1	0	0	0	0
scaffold_14	LRFN2	0	1	0	0	0	0
scaffold_0	LRP5	0	0	1	1	1	0
scaffold_8	LRP6	0	0	1	0	0	0
scaffold_29	LRRC75B	1	1	0	0	0	0
scaffold_0	LTBP2	0	0	1	0	0	0
scaffold_100	LUZP1	0	0	0	1	1	0
scaffold_43	MACROD2	1	0	0	0	0	0
scaffold_55	MAD2L2	0	0	1	0	0	0
scaffold_78	MAP2K6	1	1	0	0	0	0
scaffold_13	MCEE	0	0	0	0	1	0
scaffold_16	MCTP1	0	0	1	0	0	0
scaffold_29	MED15	1	0	0	0	0	0
scaffold_74	MELK	0	0	0	1	1	0
scaffold_55	MFN2	0	0	0	0	0	1
scaffold_55	MIIP	0	0	0	0	0	1
scaffold_60	MILR1	1	0	0	0	0	0
scaffold_60	MMD	1	0	0	0	0	0
scaffold_13	MPHOSPH10	0	0	0	0	1	0
scaffold_74	MSH3	0	0	0	1	1	0
scaffold_55	MTHFR	0	0	1	0	0	0
scaffold_55	MTOR	0	0	1	0	0	0
scaffold_9	MTTP	0	0	0	0	0	1
scaffold_64	MYCBP	0	0	0	1	1	0

scaffold_6	MYO1B	0	1	0	0	0	0
scaffold_34	NBEA	0	1	0	0	0	0
scaffold_10	NDUFB8	0	1	0	0	0	0
scaffold_0	NFKBIA	1	0	0	0	0	0
scaffold_44	NHSL2	1	0	0	0	0	0
scaffold_67	NKX6-2	0	0	0	1	1	0
scaffold_148	NME6	0	0	0	1	1	0
scaffold_58	NMNAT2	0	0	1	0	0	0
scaffold_60	NOG	1	0	0	0	0	0
scaffold_78	NOL11	1	1	0	0	0	0
scaffold_0	NOVA1	0	0	1	0	0	0
scaffold_0	NRXN3	0	1	0	0	0	0
scaffold_44	OCRL	1	0	0	0	0	0
scaffold_148	OPN1SW	0	0	0	1	1	0
scaffold_21	OSMR	0	1	0	0	0	0
scaffold_74	PAX5	0	0	0	1	1	0
scaffold_60	PCTP	1	0	0	0	0	0
scaffold_60	PECAM1	1	1	0	0	0	0
scaffold_41	PHF6	0	1	0	0	0	0
scaffold_78	PITPNC1	1	1	0	0	0	1
scaffold_0	PLA2G4F	0	1	0	0	0	0
scaffold_55	PLOD1	0	0	0	0	0	1
scaffold_60	POLG2	1	0	0	0	0	0
scaffold_29	POP5	0	0	0	1	0	0
scaffold_100	PPP1R8	0	0	0	1	1	0
scaffold_78	PRKAR1A	1	1	0	0	0	0
scaffold_78	PRKCA	1	1	0	0	0	1
scaffold_21	PRLR	0	1	0	0	0	0
scaffold_80	PROKR1	0	0	1	0	0	0
scaffold_46	PRUNE2	0	1	0	0	0	0
scaffold_78	PSMD12	1	1	0	0	0	1
scaffold_100	PTAFR	0	0	0	1	1	0
scaffold_55	PTCHD2	0	0	1	0	0	0
scaffold_16	PTPRD	0	1	0	0	0	0
scaffold_60	RAB11FIP4	1	1	0	0	0	0
scaffold_29	RAB36	1	0	0	0	0	0
scaffold_0	RALGAPA1	1	0	0	0	0	0
scaffold_56	RASA2	0	1	0	0	0	0
scaffold_74	RASGRF2	0	0	0	1	1	0
scaffold_16	RFX3	0	1	0	0	0	0
scaffold_78	RGS9	1	1	0	0	0	0

scaffold_60	RHBDL3	1	0	0	0	0	0
scaffold_60	RHOT1	1	0	0	0	0	0
scaffold_100	RLF	0	0	0	1	1	0
scaffold_29	RNF10	0	0	0	1	0	0
scaffold_60	RNF135	1	0	0	0	0	0
scaffold_7	RNPC3	0	1	0	0	0	0
scaffold_30	ROBO1	0	1	0	0	0	0
scaffold_100	RPA2	0	0	0	1	1	0
scaffold_104	RPS11	0	0	1	0	0	0
scaffold_64	RRAGC	0	0	0	1	1	0
scaffold_29	RSPH14	1	0	0	0	0	0
scaffold_1	SASH1	1	1	0	0	0	0
scaffold_71	SCAMP1	0	1	0	0	0	0
scaffold_60	SCPEP1	1	0	0	0	0	0
scaffold_78	SLC16A6	1	1	0	0	0	0
scaffold_29	SLC5A1	1	1	0	0	0	0
scaffold_58	SMG7	0	0	1	0	0	0
scaffold_104	SMPD1	0	0	1	0	0	0
scaffold_29	SMPD4	1	0	0	0	0	0
scaffold_100	SMPDL3B	0	0	0	1	1	0
scaffold_60	SMURF2	1	1	0	0	0	0
scaffold_29	SNRPD3	1	1	0	0	0	0
scaffold_48	SOX5	0	1	0	0	0	0
scaffold_29	SPECC1L	1	0	0	0	0	0
scaffold_100	STX12	0	0	0	1	1	0
scaffold_60	STXBP4	1	0	0	0	0	0
scaffold_40	SUPT6H	0	1	0	0	0	0
scaffold_60	SUZ12	1	0	0	0	0	0
scaffold_8	TBXAS1	0	1	0	0	0	0
scaffold_60	TEX2	1	1	0	0	0	0
scaffold_60	TMEM100	1	0	0	0	0	0
scaffold_54	TMEM38B	0	0	0	0	1	0
scaffold_23	TMPRSS7	0	0	0	0	1	0
scaffold_55	TNFRSF1B	0	0	0	0	0	1
scaffold_55	TNFRSF8	0	0	0	0	0	1
scaffold_148	TNPO3	0	0	0	1	1	0
scaffold_60	TOM1L1	1	0	0	0	0	0
scaffold_3	TRA2A	0	0	1	0	0	0
scaffold_60	TRIM25	1	0	0	0	0	0
scaffold_9	TRMT10A	0	0	0	0	0	1
scaffold_13	TRPM1	0	0	0	0	1	0

scaffold_63	TRPM8	0	1	0	0	0	0
scaffold_8	TTC26	1	0	0	0	0	0
scaffold_0	TTC7B	0	0	1	0	0	0
scaffold_55	UBIAD1	0	0	1	0	0	0
scaffold_29	UPB1	1	0	0	0	0	0
scaffold_60	UTP6	1	1	0	0	0	0
scaffold_10	VCL	0	1	0	0	0	0
scaffold_55	VPS13D	0	0	0	0	0	1
scaffold_78	WIPI1	1	1	0	0	0	0
scaffold_100	XKR8	0	0	0	1	1	0
scaffold_29	YWHAH	1	1	0	0	0	0
scaffold_74	ZCCHC7	0	0	0	1	1	0
scaffold_41	ZIC3	0	1	0	0	0	0
scaffold_100	ZMPSTE24	0	0	0	1	1	0
scaffold_0	ZNF106	0	0	0	0	1	0
scaffold_60	ZNF207	1	0	0	0	0	0
scaffold_0	ZNF770	0	1	0	0	0	0

Supplementary Table 9. Results from Gene Ontology and KEGG pathway enrichment analysis. Only significant results are shown.

**a) GO categories**<br>GO category name	GO category ID	ontology	p	FDR	comparison
calcium channel activity	GO:0005262	MF	5.59E-07	0.00680	*corone-cornix*
calcium ion transport	GO:0006816	BP	8.31E-06	0.03716	*corone-cornix*
calcium ion transmembrane transport	GO:0070588	BP	9.17E-06	0.03716	*corone-cornix*
calcium channel activity	GO:0005262	MF	8.20E-09	0.00010	*corone-cornix & cornix-orientalis*
calcium ion transport	GO:0006816	BP	4.07E-08	0.00012	*corone-cornix & cornix-orientalis*
voltage-gated ion channel activity	GO:0005244	MF	4.32E-08	0.00012	*corone-cornix & cornix-orientalis*
calcium ion transmembrane transport	GO:0070588	BP	4.32E-08	0.00012	*corone-cornix & cornix-orientalis*
regulation of ion transmembrane transport	GO:0034765	BP	4.86E-08	0.00012	*corone-cornix & cornix-orientalis*
voltage-gated calcium channel complex	GO:0005891	CC	1.17E-07	0.00024	*corone-cornix & cornix-orientalis*
voltage-gated calcium channel activity	GO:0005245	MF	5.80E-07	0.00101	*corone-cornix & cornix-orientalis*
ion transport	GO:0006811	BP	8.40E-06	0.01277	*corone-cornix & cornix-orientalis*
transport	GO:0006810	BP	1.72E-05	0.02326	*corone-cornix & cornix-orientalis*
**b) KEGG pathways**<br>KEGG pathway name	KEGG pathway ID		p	FDR	comparison
Cardiac muscle contraction	4260		1.55E-07	0.00002	*corone-cornix & cornix-orientalis*
MAPK signaling pathway	4010		3.78E-06	0.00029	*corone-cornix & cornix-orientalis*

Supplementary Table 10. Comparison of outlier statistics across the three contact zones.

statistic	*corone-cornix* (South)	*cornix-orientalis*	*orientalis-pectoralis*
**% contact peaks / shared peaks**	40%	24 %	30%
**peak clustering (Z-score runs test)**	-104.14	-53.20	-79.80
**nr. genes flagged (in outlier windows)**	96	58	64

Supplementary Table 11. Correlation coefficients between genome wide estimates of F_{ST} and d_{xy} for allopatric controls and hybrid zones. The lower triangle represents correlations between F_{ST} and the upper triangle correlations between d_{xy}. For the hybrid zones correlations of differentiation landscapes are shown both for absolute differentiation and net differentiation as $F_{ST}/\Delta F_{ST}$.

Allopatric

GroupID	cnx3 – cnx2	ori1 – ori3	cor1 – cor2	cor1 – ori2	*C. brachyrhynchos* - cor1
**cnx3 – cnx2**	NA	0.97	0.99	0.97	0.85
**ori1 – ori3**	0.12	NA	0.97	0.97	0.87
**cor1 – cor2**	0.21	0.25	NA	0.98	0.85
**cor1 – ori2**	0.25	0.59	0.67	NA	0.85
***C. brachyrhynchos* - cor1**	0.2	0.18	0.53	0.55	NA

Hybrid zones

GroupID	cor2 – cnx1	ori3 – pec1	cnx4 – ori1
**cor2 – cnx1**	NA	0.97/0.66	0.99/0.71
**ori3 – pec1**	0.04	NA	0.97/0.73
**cnx4 – ori1**	0.15	0.07	NA

Supplementary Table 12. Effect of sub sampling on estimates of diversity statistics (above) and linkage disequilibrium (below).

Population	Watterson's θ	Л	Tajima's D	Fay and Wu's H	Fu & Li's D	Number of individuals used
**cnx1**	0.0017	0.0017	-0.5142	0.7397	-1.0980	3
	0.0025	0.0019	-1.1909	0.5942	-2.0934	5
	0.0025	0.0017	-1.4313	0.3763	-2.6359	10
	0.0032	0.0017	-1.8200	0.3081	-3.8590	15
**cor2**	0.0014	0.0013	-0.3841	0.6899	-0.8723	3
	0.0016	0.0013	-0.6510	0.5506	-1.0538	5
	0.0018	0.0014	-0.9412	0.4068	-1.2661	10
	0.0019	0.0013	-1.0660	0.3717	-1.2780	15
**cor1**	0.0012	0.0012	-0.0571	0.4226	-0.2895	3
	0.0012	0.0012	-0.0690	0.3796	-0.1472	5
	0.0013	0.0012	-0.1148	0.3427	0.0674	10
	0.0012	0.0012	-0.1492	0.3642	0.2575	15

LD Estimates

Population	LD (r^2) mean	Number of individuals used
**cnx1**	0.2311	3
	0.1539	5
	0.1047	10
	0.0781	14
**cor2**	0.2412	3
	0.1588	5
	0.0939	10
	0.0729	15
**cor1**	0.2618	3
	0.2012	5
	0.1296	10
	0.1089	15

Supplementary Table 13. List of focal population comparisons used for genome scan in 50kb outlier windows; including target populations across contact zones with phenotypic transition and allopatric control population comparisons spanning a broad range of genomic differentiation (consult **Fig. 1** of the main manuscript for population codes).

	**Population comparison**	**Mean Fst autosomes/gonosomes**
**target comparisons contact zones**	cor2 – cnx1	0.03/0.04
	ori3 – pec1	0.04/0.06
	cnx4 – ori1	0.05/0.09
**control comparisons**	cnx3* – cnx2	0.02/0.04
	ori1 – ori3	0.07/0.13
	cor1 – cor2	0.10/0.19
	cor1 – ori2	0.25/0.51
	C. brachyrhynchos - cor1	0.27/0.39

*Swedish population only to maximize spatial distance – results qualitatively the same if Swedish and Polish populations combined.

Supplementary Table 14. The number of singleton and adjacent outlier 5 and 10 kb windows that overlap with the 50 kb outlier windows.

Contact zone comparisons	Window size	Overlap with 50 kb window	99th percentile		99.9th percentile		
			Singleton	Adjacent	Total number remaining	Singleton	Adjacent
**Contact zone 1: cor2 – cnx1**	5 kb window	match	17.5%	82.5%	497	14.9%	85.1%
		no match	66.1%	33.9%	289	100.0%	0.0%
	10 kb window	match	15.8%	84.2%	292	3.7%	96.3%
		no match	63.8%	36.2%	105	0.0%	0.0%
**Contact zone 2: cnx4 – ori1**	5 kb window	match	38.8%	61.2%	139	55.9%	44.1%
		no match	71.8%	28.2%	369	100.0%	0.0%
	10 kb window	match	43.2%	56.8%	88	27.3%	72.7%
		no match	73.6%	26.4%	174	100.0%	0.0%
**Contact zone 3: ori3 – pec1**	5 kb window	match	17.1%	82.9%	269	4.3%	95.7%
		no match	77.2%	22.8%	272	0.0%	0.0%
	10 kb window	match	18.2%	81.8%	154	4.0%	96.0%
		no match	78.6%	21.4%	126	0.0%	0.0%

Supplementary Note 1

Nomenclature

Delineation of taxonomic status within the Eurasian crow group *Corvus (corone) spp.* is contentious [4–6]. Most authors have treated (a subset of) the taxa as geographical races of a *Formenkreis* [7], comparable in meaning to the modern use of the term superspecies [8–11]. On rare occasions, the semi-species concept has been used (*C. [corone] corone, C. [corone] cornix* [12]. The case has also been made for re-elevation of some forms to full species status (*C. corone* and *C. cornix)* after their original description as species by Linnaeus [13], largely based on differences in plumage colouration and evidence for restricted gene flow between specific groups [4]. This latter concept is currently followed by many ornithological bodies (e.g. the International Ornithological Congress, see http://www.worldbirdnames.org/bow/crows/, and the Handbook of the Birds of the World, see http://www.hbw.com/species/hooded-crow-corvus-cornix, both accessed 8/10/2015). However, such clear cut delineation by plumage colouration conflicts with phylogenetic [5] and population genomic evidence of population history [2,14]. It is also apparent from this study on the level of the whole genome for populations with hooded phenotypes including *C. (c.) cornix*, *pallescens* and *pectoralis*. *C. pallescens* is sometimes recognized as a race of the *cornix* on the basis of smaller size, restricted geographical distribution and slightly lighter grey pigmentation [4]. *Pectoralis* had been treated as a separate species *C. pectoralis* (formerly called *C. torquatus*) [7,15], but was recently suggested to be nested within the species complex [6]. McCarthy [16] and Blotzheim et al. [17] recognize a parapatric contact zone between *pectoralis* and *orientalis*. Information for this area is scarce and direct evidence for hybridization is as of yet missing.

For the purpose of this manuscript (and following own recent practice [2,14]) we treat population samples from any geographical area that has at some point been recognized as taxonomically distinct with uncertainty regarding to their taxonomic status. As nominate form we choose *corone* following Meise [7] inserted in round brackets: *Corvus (corone) spp.* This approach recognizes the apparent difficulty in attributing a definite taxonomic level and in delineating hierarchical relationships between all previously described geographical forms appropriately. Samples include the American crow which together with the Northwestern Crow (*Corvus caurinus*) form the (reciprocally non-monophyletic [6]) sister clade to the *C. (corone) spp.*

complex [18].

Inclusion of the American crow

American and Eurasian crows are estimated to have separated 2.99 million years ago [19]. Assuming effective population sizes in the range of of 100,000-300,000 for populations of the species complex [12] and MSMC analyses (this study) and a generation time of approx. 6 years (this study) a split time of three million years corresponds to 1.7-5 Ne generations. Assuming that after 4-7 Ne generations still only 50% of loci show reciprocal monophyly [20] this suggests that the inclusion of the American crow in the population genetic framework is warranted. It was further supported by the fact that 11.8 % of polymorphism in Eurasian crow populations are still shared with the American crow population.

Sampling permissions

Sampling permissions were granted by *Junta de Castilla León* (Ref: CML/mjg, Expte: EP/LE/410/2010) in Spain, by *Regierungspräsidium Freiburg* (Aktenzeichen: 55-8852.15) in Germany, and by *Jordbruksverket* (Dnr 30-1326/10) in Sweden, *the Ministry of Environment of Khabarovsk krai* (permission № H-058/2009) and the *Government of Jewish Oblast, Department of Environment*, permission № 6 of 27.05.2009 in Russia.

Sample quality

Two of the *C. c. pectoralis* samples were obtained from toe pads of museum specimens collected during the 1920s. Sequencing libraries for these samples were directly produced from DNA extractions without further fragmentation. To assess the potential contribution of post-mortem DNA damage which could confound the population genetic analyses, we quantified cytosine deamination at read ends using PMDTools [21]. Visual inspection of the frequency distribution of PMD scores did not reflect any differences between museum specimens and freshly collected samples, suggesting no substantial post-mortem DNA degradation.

Sample sizes

For all analyses requiring *a priori* population classification, samples from locations with less than three individuals were grouped with adjacent, closely related populations. Sub-sampling of large population samples to 15, 10 and 3 individuals (30, 20, 6 chromosomes respectively)

indicated that key summary statistics such as Watterson's θ and Л remained stable to a set minimum of three, while others like Tajima's D, Fay and Wu's H and Fu and Li's D were inflated with only three individuals (see **Supplementary Table 11)**. Trading off fine-scale population structure to sample size, we still accepted two populations with <5 individuals (ori2, pec1 see **Supplementary Table 2**). Estimates of linkage disequilibrium, r^2, (see below) were inflated at <15 individuals which prompted us to sub-sample to equal sample sizes for population comparisons (see below).

Sex determination

Sex was determined molecularly following Griffith et al. [22] or where DNA was depleted for genomic library preparations on the basis of sequencing coverage. In the latter approach, an individual was scored as a male if the average coverage per 50 kb windows was equal between autosomes and Z-chromosomes, and as a female if the sequencing coverage of the Z-chromosome was half of the autosomal coverage as by visual inspection (see **Supplementary Fig. 6** for an example). The two approaches were in agreement in all 76 cases where both were used in a blind test. This suggests that visual inspection of coverage across chromosomes is as reliable as inspection of gel images.

Geographic distribution map

Shapefiles with the geographic distribution of the species complex were received from BirdLife International (via the form at http://www.birdlife.org/datazone/info/spcdownload, accessed at 09/10/2015). Because BirdLife International considers *Corvus (corone) corone*, *cornix*, and *orientalis* as a single species, the distribution map could only be downloaded as a single shapefile (a separate file was obtained for *pectoralis*). We next split the distribution into a separate shapefile for each of the three subspecies as well as files for the hybrid zones. With respect to the hybrid zone between *corone* and *cornix*, the Scottish part was drawn according to [23], and the Danish and central European (Germany to Italy) parts according to [24]. The hybrid zone between *cornix* and *orientalis* was drawn according to [25]. There were some discrepancies between the Birdlife distribution and distributions from other sources, most notably in central Asia where e.g. Blinov [25] shows a large area without any crows occurring, whereas the Birdlife distribution includes these areas; we adhered to the Birdlife distribution as much as possible and extended the putative position of the hybrid zone in south-central Asia (where the position and extent is unclear over a larger range) where necessary. Furthermore, we extended the hybrid zone into eastern Northern Ireland where hybrid phenotypes prevail

(personal observation JBW Wolf).

Isolation by distance

The geographic distance between populations was calculated using the function spDistsN1 from the R package *sp* [26]. A Mantel test as implemented in the R package ecodist [27] was used to assess statistical significance of the correlation between geographic distance and genetic distance, while controlling for pseudo-replication among all possible population comparisons. For population pools from different sampling locations (cnx3, cnx2, cnx4, ori1), the average longitude and latitude values weighted by sample size were used as geographic location. Following [28], $F_{ST}/1\text{-}F_{ST}$ was used as genetic distance. In the main text, we report the correlation for entire nuclear genome; the correlations for sex chromosome and autosomes were near identical (r=0.47/0.46, p=0.007/0.008).

Mitochondrial phylogeny

We downloaded the published mitochondrial genome of the American crow (*Corvus brachyrhynchos,* GenBank: KR072661.1) and aligned eighty-one complete *corone/cornix/orientalis/pectoralis* crow mitochondrial genomes using the program CSA [29], a multiple sequence alignment algorithm that rotates circular DNA to match cut sites across multiple circular genomes. Subsequent to rotation, a second round of multiple sequence alignment, specifically to align gap regions, was performed with MUSCLE [30]. This rotation and realignment allowed us to use the current annotation of the American crow mitochondrial genome to partition the data for subsequent maximum-likelihood phylogenetic analyses. Our data was partitioned for 1st and 2nd codon positions and 3rd codon position of coding regions separately, and combined tRNA and rRNA regions. The GTR+Γ substitution model was implemented for 1000 bootstrap replicates. Phylogenetic trees were reconstructed using maximum-likelihood (ML) in RAxML 7.0.4 [31], using the rapid bootstrapping algorithm [32].

Repeat annotation

We performed *de-novo* prediction of crow-specific repeats by analyzing the hooded crow assembly using RepeatModeler (version 1.0.5; http://www.repeatmasker.org/RepeatModeler.html). RepeatModeler identifies and models repeats by employing the complementary programs RECON (version 1.07; [33]), RepeatScout (version 1.0.5; [34]), and Tandem Repeats Finder (version 4.0.4; [35]). The resulting library of repeat candidates was manually inspected according to standard procedures [36]. We focused on young repeat families

(i.e., low sequence divergence among copies), all of which were classified by RepeatModeler as belonging to the long terminal repeat (LTR) retrotransposons. We BLASTn-searched [37] each of these repeat candidates against the hooded crow assembly, extracted maximally 50 of the best hits including 1-kb flanks, and aligned the BLASTn hits of each repeat candidate using MAFFT [38]. From each of these alignments, we constructed a manually curated consensus sequence that was considered to be complete only if the corresponding BLASTn hits were flanked by single-copy sequence at its 5' and 3' ends. The resultant repeat library comprised 29 complete LTR subfamilies and 80 potentially incomplete repeat consensus sequences. We then combined these crow repeats with chicken and zebra finch repeat consensus sequences available in RepBase (http://www.girinst.org/repbase/index.html) and annotated repetitive elements in the hooded crow assembly via RepeatMasker (version 3.2.9; http://www.repeatmasker.org/RMDownload.html).

Multiple sequentially Markovian coalescent (MSMC)

The multiple sequentially Markovian coalescent (MSMC) approach [39] was run for one individual at a time and therefore used unphased data (phasing is only needed when using more than one individual). We used the 100 largest scaffolds and excluded scaffolds inferred to be sex-linked. Since performance of sequential Markovian coalescent methods has been shown to be negatively affected when coverage is below 20x [40], we only used individuals with a mean coverage of 20x or higher. Input files and a mask file for regions with excessively low and high coverage were generated using scripts provided along with the MSMC program (https://github.com/stschiff/msmc-tools). The default time period scheme of "10×1 + 15×2" was used.

In order to rescale time and population size, we used a mutation rate estimate of 3.18×10^{-9} per generation as estimated for *C. brachyrhynchos* by [41], and inferred generation time as follows. The generation time was estimated at 5.79 years using the formula $T = \alpha + s / (\lambda - s)$ [42], where T is generation time, α is age at first reproduction in years, s is yearly adult survival (we used α=3 following [17] and s= 0.736 following Møller [43], and λ is the population growth rate. For the latter, we used a value of 1.0 (no growth), since we are interested in the long-term generation time (with a yearly 1% population growth rate, the generation time would be 5.69).

Another method of estimating generation time is to simply multiply the age of sexual maturity

by two (see Nadachowska-Brzyska et al. [41]), which would result in the fairly similar estimate of T = 6 (using three years for the age of sexual maturity following [17] and The Animal Ageing and Longevity Database http://genomics.senescence.info/species/entry.php?species=Corvus_corone). With a generation time of 5.79, the above mentioned mutation rate estimate for *C. brachyrhynchos* per generation corresponds to a per year mutation rate of 0.55 x 10^{-9}, which is lower than the estimate commonly used for birds (3.6 x 10^{-9} per year [44], 1.2-1.5 x 10^{-9} [45]). The time estimates reported in the main text therefore most likely constitute an upper (long time scale) bound.

We converted scaled times and population sizes as specified on https://github.com/stschiff/msmc/blob/master/guide.md. That is, to convert scaled times as output by MSMC to time in years, we multiplied by the per year mutation rate. To convert scaled population sizes (1 / coalescence rate) to population sizes, we divided by twice the per generation mutation rate.

F-statistics

To assess robustness of genotype-based F_{ST} estimates from *HierFstat* described in the main text, we additionally used methods specifically designed for low-to-medium-coverage sequencing data. Using ANGSD, the unfolded site frequency spectrum (SFS) was estimated as described above. Using this maximum-likelihood estimate of the SFS as a prior in an Empirical Bayes approach [46], the posterior probability of all possible allele frequencies at each site was then computed using the software package *NGStools* [47,48]. Expectations of the number of variable sites and fixed differences between lineages were then estimated as the sum across sites of the probability of each site to be variable as previously proposed. Finally, the posterior expectation of the sample allele frequencies was calculated as the basis for further analysis of genetic variation within and between lineages.

F_{ST} was estimated with a method-of-moments estimator [49] based upon the sample allele frequency posterior probabilities of the 2D-SFS. These were highly correlated with the genotype-based estimates inferred by *HierFstat* for a selection of eight pairwise comparisons (Pearson’s correlation coefficient: range r = 0.81-0.93).

Genome scans

Window-based. To isolate candidate genomic regions under selection, we followed the basic

logic of traditional genome scans screening for signals of elevated genetic differentiation [50] on the basis of non-overlapping windows of predefined size. Previous work has shown the suitability of 50 kb windows to asses broad-scale patterns of genetic heterogeneity in genetic differentiation measures, such as F_{ST} averaging across local variance [2]. To test for the suitability of smaller window sizes potentially highlighting local genomic regions that would go undetected when averaging across 50 kb intervals, we additionally calculated summary statistics for windows sizes of 5 kb and 10 kb. We compared smaller window sizes for consistency to 50 kb outlier windows (see below for how they are determined) using the rationale that 1) smaller windows should likewise flag large, cohesive genomic regions of significantly elevated F_{ST} indicated by runs of 50 kb outlier windows (see below) and 2) may add few additional windows of interest.

In the following we report results for outlier windows at the 99th percentile threshold in the European hybrid zone, though the pattern holds for the other two contact zones (**Supplementary Table 14**). While 74% of the 10 kb outlier windows overlapped with broad-scale 50 kb peaks, overlap was reduced to 63% for the smaller 5 kb windows, with an even greater reduction for the two other contact zones. A reduction in overlap with decreasing window size suggests an increase in false positives (noise). Moreover, the proportion of aggregate outlier windows, suggestive of a true positive signal, was substantially higher for overlapping windows of both small size classes compared to singletons outlier windows, suggestive of false negative noise signals or additional true positive signals not captured in 50 kb windows. When a less permissive 99.9[th] percentile threshold was used, thus considering only extreme outlier windows overlapping the 50 kb peaks, the degree of singleton windows was reduced to less than 20% for 5 kb windows and less than 10% for 10 kb windows. Few windows outside of the prominent peak regions defined by the 50 kb windows showed extreme F_{ST} values at this threshold for any small size class. Importantly, hundred(s) of singleton outlier regions flagged by both small size classes are biologically unrealistic and most likely reflect noise in the form of sampling variance. Marked F_{ST} outlier peaks are only expected for traits with simple genetic architectures (single to few genes) under strong, recent selection. Selection on a polygenic architecture, which would in principle allow many signals across the genome, is not expected to leave strong signals in genome scans [51,52]. Overall, a 50 kb window size therefore seems better suited to study broad-scale patterns of genome-wide differentiation arising by long-term linked selection and may pick up few, strong and recent selection events. The following description of window-based analyses and results in the main

text therefore refer to a window size of 50 kb. Finer-scale patterns were studied using local phylogenies (SOM-HMM) and single outlier SNPs (see below).

To enable direct comparison of F_{ST} windows between all pairwise comparisons with different degrees of mean genome-wide differentiation, F_{ST} values were Z-transformed (denoted as F_{ST}'). The transformation was conducted separately for autosomes and the sex chromosome because of the observed, and theoretically expected, difference in the degree of differentiation (due to differences in N_e). This transformation balanced original differences in the shape of the F_{ST} distribution between population pairs (right skew for less differentiated population pairs) and approximated a standard normal Gaussian distribution N(0;1) (**Supplementary Fig. 7**). Windows with F_{ST}' values exceeding a threshold given by the 99th percentile of the distribution (> 2.3 standard deviations) were considered 'outliers' potentially under the influence of selection. To test for non-random distribution of outlier windows across the genome, windows were coded as a binary vector (0: 1-99th percentile, 1: >99th percentile). The Runs test statistic [53] implemented as the runs.test method in the R *tseries* package [54] was then used to test for under-mixing of the two categories (clustering of outliers) relative to the expectation of random permutation. The test statistic is defined as $Z = (R_{obs}-R_{exp})/s_R$ where R_{obs} is the observed number of runs (consecutive 0 or 1 values), R_{exp} is the expected number of runs and s_R is the standard deviation of the number of runs. R_{exp} and s_R are computed as: $R_{exp}=(2n1n2)/((n1+n2)+1=$; $s^2_R=(2n1n2(2n1n2-n1-n2))/((n1+n2)^2(n1+n2-1))$ with n1 and n2 denoting the number of consecutive positive and negative values in series. The test rejects the null hypothesis of random mixing if $|Z|=Z1-\alpha/2$. For large sample sizes, as in our case (n1>10, n2>10) the test statistic converges to a standard normal distribution. The test statistic Z is negative for under-mixing (i.e. outlier windows cluster more than expected by chance) and positive for over-mixing (i.e. outlier windows cluster less than expected by chance).

As another measure of the non-randomness of the distribution of F_{ST} values along the genome, we assessed the degree of autocorrelation between adjacent windows. Autocorrelation of F_{ST} values across windows was calculated for each scaffold and pairwise comparison with Moran's I. To do this, we computed a distance matrix for all windows within a scaffold and used this along with the corresponding F_{ST} values for each window as arguments in the Moran.I function from the *ape* R package [55], which follows the method by Gittleman & Knot [56].

To infer evolutionary process from the heterogeneous pattern of broad scale F_{ST}', we took a comparative approach as has been suggested previously [e.g. ,57–61]. Our focal comparisons were between populations with evidence for gene flow and/or transition in pigmentation phenotype including the parapatric south-eastern Russian *C. c. orientalis* population with collared crows (ori3-pec1) and populations surrounding the European and Siberian hybrid zones (cor2-cnx1 and cnx4-ori1 respectively, see **Fig. 1**). For the European hybrid zone, we focused on the comparison between the German carrion crow population (cor2) and the Italian hooded crow population (cnx1) rather than Swedish or Polish hooded crow populations (cnx3), which had been studied previously at genome-scale resolution [2]. To assess whether outlier peaks were driven by processes unique to these phenotypically divergent comparisons (e.g. divergent selection against gene flow) or by common shared selection pressures (e.g. background selection) we contrasted their genomic F_{ST} profiles to a set of five control comparisons. These controls, listed in **Supplementary Table 12**, were chosen in order:

1) to include comparisons within and between subspecies,

2) to include comparisons across the entire species range (broad geographic representation),

3) and minimize the probability of recently occurring gene flow by choosing geographically distant populations ('allopatry'),

4) to span a broad range of mean genome-wide differentiation, allowing to study the built-up of differentiation islands with increasing drift,

5) to minimize pseudo-replication by making sure that populations used in any of the focal comparisons would be used at most once, and

6) to control for influence of phenotype focusing on population comparisons of the same pigmentation phenotype (no phenotypic contrast).

Based on these criteria control population comparisons were chosen as follows:

1) A comparison between two hooded crow populations not used in any of the target comparisons (cnx2 – cnx3). The low levels of genome-wide F_{ST} make it difficult to separate recent gene flow from recent shared ancestry. To minimize the potential influence of current gene flow, we maximized geographic distance by restricting the comparison to cnx2 with only

the Swedish cnx3 population. The fact that all hooded crow populations seem to share recent common ancestry (see **Fig. 1**) supports the idea that low levels of F_{ST} are not predominantly a function of ongoing gene flow but rather of recent ancestry.

2) Comparisons between three all-black populations within and between *C. c. corone* and *C. c. orientalis* spanning a range of genome-wide differentiation (**Supplementary Table 12**).

3) The most divergent comparison of the American crow, *C. brachyrhynchos,* with the species complex. Inclusion of American crow into the population genetic analysis is justified as these species still share 11.6% of segregating variation. Assuming three million years of divergence [19], a generation time of 5.79 years and an effective population size of 100,000 (this study) - 200,000 [12] would put the time to the most recent common ancestor at around 3-6 N_e generations, which indicated that lineage sorting is also theoretically expected to still be incomplete. Yet, given more recent common ancestry of the Eurasian species complex, levels of differentiation should be similar for comparisons of the American crow with any of the sampled Eurasian crow populations (unless influenced by extreme local demographic perturbations such as bottlenecks). Accordingly, heterogeneity in differentiation across the genome was observed to be similar among comparisons ($r_{Pearson}$ scaled F_{ST} range: 0.77-0.97, $p_{all}<0.001$). The only exception was the Russian *ori2* population, which showed lower correlation values ($r_{Pearson}$ scaled F_{ST} range: 0.61-0.75, $p_{all}<0.001$), potentially due to its low sample size. In the main text, we report the comparison of the American crow with the Spanish population *cor1* satisfying the above criteria. Due to the high correlation of F_{ST} profiles, a different choice of Eurasian populations should produce qualitatively similar results. This was explicitly tested for the comparisons between American Crow with all hooded crows pooled. This constitutes the most conservative contrast, since it integrates potential signatures of sweeps during the recent history of hooded crows, which may contribute to local differentiation caused by reduced diversity, but not by locally reduced gene flow with the American crow.

In a first step, we determined outlier windows for all controls at the 99th percentile of the Z-transformed F_{ST} distribution (F_{ST}') and quantified number of peaks and peak width (in number of adjacent windows). These peaks can be regarded as background heterogeneity arising through processes other than divergent selection across hybrid zones. We then characterized outlier windows of the focal comparisons in the same way. In addition, we subtracted the maximum value of orthologous windows in the controls from each of the focal comparisons (**Fig. 2**) and determined outlier windows at the 99th percentile for this statistic (called Δ F_{ST}'

hereafter, cf. delta divergence in Roesti et al. (2014) [62]). Windows classified as outliers for F_{ST}', but not $\Delta F_{ST}'$, are interpreted as genomic regions subject to shared selection pressures (e.g. background selection in areas of low recombination) across the entire species complex independent of specific evolutionary processes acting on any of the target populations. Windows classified as outliers by both approaches were considered to be unique to each focal comparison in genomic position (no peak in controls, but peak in focal population) and/or relative amplitude (also peak in outlier, but with comparatively lower standardized peak height). These 'unique' outliers were investigated in more detail for gene content, and were contrasted to background genome-wide non-peak regions as well as common peaks for a set of informative summary statistics such as nucleotide diversity (π), net nucleotide divergence (D_{xy}), Fay's H, and haplotype statistics (r^2, iHH, iHS, nSL and XP-EHH; **Supplementary Table 6,7**).

Several summary statistics support a signal of selection within the unique remaining windows across the *corone-cornix* and *orientalis-pectoralis* contact zones. These unique outlier regions showed significant departures from background regions, for instance reduced nucleotide diversity (π), and divergence (D_{xy}), and longer haplotype blocks (iHH), increased linkage (r^2), and increased branch specific selection (PBS) (**Supplementary Table 6**). However this broad pattern of significance is not found within the smaller peak regions in the *cornix-orientalis* contact zone, suggesting weakened localized selection generating or even maintaining peaks (**Supplementary Table 7)**. When comparing the remaining peaks to the shared peaks detected in the allopatric controls, the number of significance differences decreased (**Supplementary Table 7**), suggesting similar selection signals. This is not altogether unsurprising, given these shared peaks could have shared selection pressures, however our remaining peaks should detect localized selection, potentially against gene flow in contact regions.

Localized phylogenetic patterns (cacti). Highly localised patterns of population divergence cannot be detected by window based methods. Hence, to complement the window based analysis, we used the HMM-SOM method implemented in Saguaro [63] to identify local phylogenetic relationships across each of the target zones of contact and phenotypic transition (cor2-cnx1, cnx4-ori1, ori3-pec1). Saguaro was run with default settings to generate 10 different cacti for each population that could be manually classified into two main classes based on their ability to distinguish the taxa.

1) European hybrid zone: cor2-cnx1

Nine out of ten cacti, covering approximately 99.97% of the genome, did not show a clear delineation by taxon. In line with previous findings, only 0.03% of the genome, approximately 4,500 variants, reflected a clear separation by taxon into hooded and carrion crows. Extensive overlap between variants found in the phylogenetically distinct genomic regions and the 99th percentile FST outlier windows, 76%, and ΔF_{ST}' remaining windows, 62%, demonstrate congruency across methods, as well as the ability of the localized phylogenetic patterns to pick up unique and smaller genomic regions missed by the window-based approaches.

2) Siberian hybrid zone: cnx4-ori1

Only one out of ten cacti, covering approximately 0.09% of the genome and containing approximately 14,669 variants, separated the taxa. Only 52% of these variants also fell within the 99th percentile F_{ST} outlier windows, indicating that differentiation across this hybrid zone is less distinct than across either of the two other contact zones (see below).

3) East-Asian contact zone: ori3-pec1

Two out of ten cacti, covering approximately 0.11% of the genome and containing 18,414 variants, clearly separated the taxa. 93% of these variants also fell within the 99th percentile F_{ST} outlier windows, including an 85% overlap with the remaining ΔF_{ST}' windows.

SNP-based analyses. In total we identified 183 fixed differences (of which two fell within exons) between cor2-cnx1, 35 fixed differences (one within an exon) between cnx4-ori1, and 1054 fixed differences (86 within exons) between ori3-pec1. Both the fixed differences found within exons between cor2-cnx1 where located in the 3'UTR of the *RGS9* gene. The fixed difference found within an exon between cor2-cnx1 was located in the gene *LRP5*. Of the 86 fixed differences found within exons between ori3-pec1, 22 where located within the gene *HNRNPR*, 8 each in *HTR1D* and *LUZP1*, 6 in *KDM1A*, 5 in *RLF*, 4 each in *STX12* and *GJA9*, 3 each in *EYA3* and LOC104694803, 2 each in *XKR8*, *LMBR1* and LOC104689048, 1 each in *CRY2, LRP5, CPNE7, PPARD, GAD1, GRHL3, PLEKHM1*, LOC104683486, *ATPIF1, DNAJC8, PTAFR, CITED4, ZMPSTE24, PPP1R8, RAP1GAP* and LOC104694723.

Substitution rate estimation

Coding regions of canonical transcripts were downloaded from Ensembl 81 for chicken (Gallus gallus) and collared flycatcher (Ficedula albicollis). Open reading frames from orthologous crow genes (orthology was inferred from NCBI annotation) were extracted from the NCBI annotation of the crow genome. These sequences were used to generate a three-way codon-based alignment with GUIDANCE-HoT [64] using PRANK's progressive alignment algorithm [65]. From 5,012 resulting reliable 1:1:1 gene-alignments, substitution rate estimates were obtained at 4-fold degenerate sites (d4) using model 0 for codons as implemented in CODEML from PAML version 4.7 [66]. Genes with d4 estimates larger than 1.5 were removed, as such high substitution rates are likely to reflect incorrectly inferred orthology or reading frame rather than biological reality.

Supplementary References

1. Zhang, G. *et al.* Comparative genomic data of the Avian Phylogenomics Project. *GigaScience* **3,** 26 (2014).
2. Poelstra, J. W. *et al.* The genomic landscape underlying phenotypic integrity in the face of gene flow in crows. *Science* **344,** 1410–1414 (2014).
3. Serre, D., Nadon, R. & Hudson, T. J. Large-scale recombination rate patterns are conserved among human populations. *Genome Res.* **15,** 1547–1552 (2005).
4. Parkin, D. T., Collinson, M., Helbig, A. J., Knox, A. G. & Sangster, G. The taxonomic status of Carrion and Hooded Crows. *Br. Birds* **96,** 274–290 (2003).
5. Haring, E., Gamauf, A. & Kryukov, A. P. Phylogeographic patterns in widespread corvid birds. *Mol. Phylogenet. Evol.* **45,** 840–862 (2007).
6. Haring, E., Däubl, B., Pinsker, W., Kryukov, A. & Gamauf, A. Genetic divergences and intraspecific variation in corvids of the genus *Corvus* (Aves: Passeriformes: Corvidae) – a first survey based on museum specimens. *J. Zool. Syst. Evol. Res.* **50,** 230–246 (2012).
7. Meise, W. Die Verbreitung der Aaskrähe (Formenkreis *Corvus corone* L.). *J. Für Ornithol.* **76,** 1–203 (1928).
8. Saino, N., Lorenzini, R., Fusco, G. & Randi, E. Genetic variability in a hybrid zone between carrion and hooded crows (*Corvus corone corone and C. c. cornix* , Passeriformes, Aves) In North-Western Italy. *Biochem. Syst. Ecol.* **20,** 605–613 (1992).
9. Kryukov, A. P. & Suzuki, H. Phylogeography of carrion, hooded, and jungle crows (Aves, Corvidae) inferred from partial sequencing of the mitochondrial DNA cytochrome b gene. *Russ. J. Genet.* **36,** 922–929 (2000).
10. Mallet, J. in *Encyclopedia of Biodiversity* 523–526 (Academic Press, 2001).
11. Haas, F. *et al.* An analysis of population genetic differentiation and genotype-phenotype association across the hybrid zone of carrion and hooded crows using microsatellites and MC1R. *Mol. Ecol.* **18,** 294–305 (2009).
12. Wolf, J. B. W. *et al.* Nucleotide divergence vs. gene expression differentiation:

comparative transcriptome sequencing in natural isolates from the carrion crow and its hybrid zone with the hooded crow. *Mol. Ecol.* **19 Suppl 1,** 162–75 (2010).

13. Linnaeus, C. *Systema Naturae per regna tria naturae, secundum classes, ordines, genera, species, cum characteribus, differentiis, synonymis, locis*. (1758).
14. Poelstra, J. W., Ellegren, H. & Wolf, J. B. W. An extensive candidate gene approach to speciation: diversity, divergence and linkage disequilibrium in candidate pigmentation genes across the European crow hybrid zone. *Heredity* **111,** 467–473 (2013).
15. Madge, S. & Burn, H. *Crows and Jays*. (A&C Black, 2010).
16. McCarthy, E. M. *Handbook of Avian Hybrids of the World*. (Oxford University Press New York, 2006).
17. Blotzheim, G. von, Bauer, K. M. & Bezzel, E. *Handbuch der Vögel Mitteleuropas*. (AULA, 1993).
18. Jønsson, K. A., Fabre, P.-H. & Irestedt, M. Brains, tools, innovation and biogeography in crows and ravens. *BMC Evol. Biol.* **12,** 72 (2012).
19. Jønsson, K. A. *et al.* A supermatrix phylogeny of corvoid passerine birds (Aves: Corvides). *Mol. Phylogenet. Evol.* **94,** 87–94 (2016).
20. Hudson, R. R., Coyne, J. A. & Huelsenbeck, J. Mathematical consequences of the genealogical species concept. *Evolution* **56,** 1557–1565 (2002).
21. Skoglund, P. *et al.* Separating endogenous ancient DNA from modern day contamination in a Siberian Neandertal. *Proc. Natl. Acad. Sci.* **111,** 2229–2234 (2014).
22. Griffith, R., Double, M. C., Orr, K. & Dawson, R. J. G. A DNA test to sex most birds. *Mol. Ecol.* **7,** 1071–1075 (1998).
23. Cook, A. Changes in Carrion-Hooded Crow hybridzone and the possible importance of climate. *Bird Study* **22,** 165–168 (1975).
24. Haas, F. & Brodin, A. The Crow *Corvus corone* hybrid zone in southern Denmark and northern Germany. *Ibis* **147,** 649–656 (2005).
25. Blinov, V. N., Blinova, T. K. & Kryukov, A. P. in *Gibridizatsiya i problema vida u pozvonochnykh* 97–117 (1993).
26. Bivand, R. S., Pebesma, E. & Gómez-Rubio, V. *Applied Spatial Data Analysis with R*.

(Springer, 2013).

27. Goslee, S. C. & Urgban, D. L. The ecodist package for dissimilarity-based analysis of ecological data. *J. Stat. Softw.* **22,** 1–19 (2007).
28. Rousset, F. Genetic differentiation and estimation of gene flow from F-statistics under isolation by distance. *Genetics* **145,** 1219–1228 (1997).
29. Fernandes, F., Pereira, L. & Freitas, A. T. CSA: An efficient algorithm to improve circular DNA multiple alignment. *BMC Bioinformatics* **10,** 230 (2009).
30. Edgar, R. C. MUSCLE: multiple sequence alignment with high accuracy and high throughput. *Nucleic Acids Res.* **32,** 1792–1797 (2004).
31. Stamatakis, A. RAxML-VI-HPC: maximum likelihood-based phylogenetic analyses with thousands of taxa and mixed models. *Bioinformatics* **22,** 2688–2690 (2006).
32. Stamatakis, A., Hoover, P. & Rougemont, J. A rapid bootstrap algorithm for the RAxML Web servers. *Syst. Biol.* **57,** 758–771 (2008).
33. Bao, Z. & Eddy, S. R. Automated de novo identification of repeat sequence families in sequenced genomes. *Genome Res.* **12,** 1269–1276 (2002).
34. Price, A. L., Jones, N. C. & Pevzner, P. A. De novo identification of repeat families in large genomes. *Bioinformatics* **21,** i351–i358 (2005).
35. Benson, G. Tandem repeats finder: a program to analyze DNA sequences. *Nucleic Acids Res.* **27,** 573–580 (1999).
36. Lavoie, C. A., Platt, R. N., Novick, P. A., Counterman, B. A. & Ray, D. A. Transposable element evolution in *Heliconius* suggests genome diversity within Lepidoptera. *Mob. DNA* **4,** 21 (2013).
37. Altschul, S. F., Gish, W., Miller, W., Myers, E. W. & Lipman, D. J. Basic local alignment search tool. *J. Mol. Biol.* **215,** 403–410 (1990).
38. Katoh, K. & Toh, H. Recent developments in the MAFFT multiple sequence alignment program. *Brief. Bioinform.* **9,** 286–298 (2008).
39. Schiffels, S. & Durbin, R. Inferring human population size and separation history from multiple genome sequences. *Nat. Genet.* **46,** 919–925 (2014).
40. Orlando, L. *et al.* Recalibrating Equus evolution using the genome sequence of an early

Middle Pleistocene horse. *Nature* **499,** 74–78 (2013).

41. Nadachowska-Brzyska, K., Li, C., Smeds, L., Zhang, G. & Ellegren, H. Temporal dynamics of avian populations during pleistocene revealed by whole-genome sequences. *Curr. Biol.* **25,** 1375–1380 (2015).
42. Niel, C. & Lebreton, J.-D. Using demographic invariants to detect overharvested bird populations from incomplete data. *Conserv. Biol.* **19,** 826–835 (2005).
43. Møller, A. P. Sociality, age at first reproduction and senescence: comparative analyses of birds. *J. Evol. Biol.* **19,** 682–689 (2006).
44. Axelsson, E., Smith, N. G. C., Sundtrom, H., Berlin, S. & Ellegren, H. Male-biased mutation rate and divergence in autosomal, Z-linked and W-linked introns of chicken and turkey. *Mol. Biol. Evol.* **21,** 1538–1547 (2004).
45. Ellegren, H. Molecular evolutionary genomics of birds. *Cytogenet. Genome Res.* **117,** 120–130 (2007).
46. Nielsen, R., Korneliussen, T., Albrechtsen, A., Li, Y. & Wang, J. SNP calling, genotype calling, and sample allele frequency estimation from new-generation sequencing data. *PLoS ONE* **7,** e37558 (2012).
47. Fumagalli, M. *et al.* Quantifying population genetic differentiation from next-generation sequencing data. *Genetics* **195,** 979–992 (2013).
48. Fumagalli, M., Vieira, F. G., Linderoth, T. & Nielsen, R. ngsTools: methods for population genetics analyses from next-generation sequencing data. *Bioinformatics* **30,** 1486–1487 (2014).
49. Reynolds, J., Weir, B. S. & Cockerham, C. C. Estimation of the coancestry coefficient: basis for a short-term genetic distance. *Genetics* **105,** 767–779 (1983).
50. Lewontin, R. C. & Krakauer, J. Distribution of gene frequency as a test of the theory of the selective neutrality of polymorphisms. *Genetics* **74,** 175–195 (1973).
51. Le Corre, V. & Kremer, A. The genetic differentiation at quantitative trait loci under local adaptation. *Mol. Ecol.* **21,** 1548–1566 (2012).
52. Rockman, M. V. The Qtn program and the alleles that matter for evolution: all that's gold does not glitter. *Evolution* **66,** 1–17 (2012).

53. Bradley, J. V. *Distribution-Free Statistical Tests*. (Prentice-Hall, 1968).
54. R Core Team. *R: A language and environment for statistical computing*. (R Foundation for Statistical Computing, 2012).
55. Paradis, E., Claude, J. & Strimmer, K. APE: analyses of phylogenetics and evolution in R language. *Bioinformatics* **20,** 289–290 (2004).
56. Gittleman, J. L. & Kot, M. Adaptation: statistics and a null model for estimating phylogenetic effects. *Syst. Biol.* **39,** 227–241 (1990).
57. Kronforst, M. R. *et al.* Hybridization reveals the evolving genomic architecture of speciation. *Cell Rep.* **5,** 666–677 (2013).
58. Martin, S. H. *et al.* Genome-wide evidence for speciation with gene flow in *Heliconius* butterflies. *Genome Res.* **23,** 1817–1828 (2013).
59. Seehausen, O. *et al.* Genomics and the origin of species. *Nat. Rev. Genet.* **15,** 176–192 (2014).
60. Feulner, P. G. D. *et al.* Genomics of divergence along a continuum of parapatric population differentiation. *PLoS Genet* **11,** e1004966 (2015).
61. Berner, D. & Salzburger, W. The genomics of organismal diversification illuminated by adaptive radiations. *Trends Genet.* **31,** 491–499
62. Roesti, M., Gavrilets, S., Hendry, A. P., Salzburger, W. & Berner, D. The genomic signature of parallel adaptation from shared genetic variation. *Mol. Ecol.* **23,** 3944–3956 (2014).
63. Zamani, N. *et al.* Unsupervised genome-wide recognition of local relationship patterns. *BMC Genomics* **14,** 347 (2013).
64. Landan, G. & Graur, D. Local reliability measures from sets of co-optimal multiple sequence alignments. *Pac. Symp. Biocomput.* 15–24 (2008).
65. Löytynoja, A. & Goldman, N. An algorithm for progressive multiple alignment of sequences with insertions. *Proc. Natl. Acad. Sci. U. S. A.* **102,** 10557–10562 (2005).
66. Yang, Z. H. PAML 4: Phylogenetic analysis by maximum likelihood. *Mol. Biol. Evol.* **24,** 1586–1591 (2007).
